# Cytogenetic profile of 1791 adult acute myeloid leukemia in India

**DOI:** 10.1186/s13039-023-00653-1

**Published:** 2023-09-16

**Authors:** Vivi M. Srivastava, Sukesh Chandran Nair, Marimuthu Sappani, Marie-Therese Manipadam, Uday P. Kulkarni, Anup J. Devasia, N. A. Fouzia, Anu Korula, Kavitha M. Lakshmi, Aby Abraham, Alok Srivastava

**Affiliations:** 1 Department of Cytogenetics, Christian Medical College, Vellore, Tamil Nadu 632004 India; 2 Department of Transfusion Medicine and Immunohaematology, Christian Medical College, Vellore, Tamil Nadu 632004 India; 3 Department of Biostatistics, Christian Medical College, Vellore, Tamil Nadu 632002 India; 4 Department of General Pathology, Christian Medical College, Vellore, Tamil Nadu 632004 India; 5 Present Address: Department of Cellular Pathology, Maidstone Hospital, Hermitage Lane, Maidstone, ME169QQ UK; 6 Department of Clinical Haematology, Christian Medical College, Vellore, 632501 Tamil Nadu India; 7 Present Address: On leave at Princess Margaret Cancer Centre, Toronto, Canada; 8 Present Address: NCCCR, Doha, Qatar

**Keywords:** Acute myeloid leukemia, Cytogenetics, Chromosomal, Frequency, Asia, Complex karyotype, Monosomal karyotype, Myelodysplasia-related, Translocation, Age

## Abstract

**Background:**

Cytogenetic analysis continues to have an important role in the management of acute myeloid leukemia (AML) because it is essential for prognostication. It is also necessary to diagnose specific categories of AML and to determine the most effective form of treatment. Reports from South Asia are few because the availability of cytogenetic services is relatively limited.

**Methods:**

We performed a retrospective analysis of the cytogenetic findings in adults with AML seen consecutively in a single centre in India. The results were categorised according to the 2022 World Health Organisation (WHO), International Consensus Classification (ICC) and European LeukemiaNet (ELN) classifications.

**Results:**

There were 1791 patients aged 18–85 years (median age 42, 1086 males). Normal karyotypes were seen in 646 (36%) patients. The 1145 (64%) abnormal karyotypes comprised 585 (32.7%) with recurrent genetic abnormalities (RGA), 403 (22.5%) with myelodysplasia-related cytogenetic abnormalities (MRC), and 157 (8.8%) with other abnormalities. There were 567 (31.7%) patients with solitary abnormalities and 299 (16.7%) with two abnormalities. Among the 279 (15.6%) patients with ≥ 3 abnormalities, 200 (11.2%) had complex karyotypes (CK) as per the WHO/ICC and 184 (10.3%), as per the ELN definition. There were 158 (8.8%) monosomal karyotypes (MK). Patients with normal karyotypes had a higher median age (45 years) than those with abnormal karyotypes (40 years, *p* < 0.001), and those with ≥ 3 abnormalities (43 years), than those with fewer abnormalities (39 years, *p* = 0.005**).** Patients with CK (WHO/ICC) and monosomal karyotypes had a median age of 48 years. Those with RGA had a lower median age (35 years, *p* < 0.001) than MRC (46 years) or other abnormalities (44 years). The t(15;17) was the most common abnormality (16.7%),followed by trisomy 8 (11.6%), monosomy 7/del 7q (9.3%), t(8;21) (7.2%), monosomy 5/del 5q (6.7%) and monosomy 17/del 17p (5.2%).

**Conclusion:**

Our findings confirm the lower age profile of AML in India and show similarities and differences with respect to the frequencies of individual abnormalities compared to the literature. The frequencies of the t(15;17), trisomy 8 and the high-risk abnormalities monosomy 7 and monosomy 5/del 5q were higher, and that of the inv(16), lower than in most reports.

**Supplementary Information:**

The online version contains supplementary material available at 10.1186/s13039-023-00653-1.

## Background

Cytogenetic analysis continues to be an important part of the work up of acute myeloid leukemia (AML) because the chromosomal constitution of a leukemia has a major impact on prognosis [1]. It is also essential for the diagnosis of two categories which are based upon the presence of specific cytogenetic abnormalities, namely, AML with recurrent genetic abnormalities (AML-RGA) and AML with myelodysplasia-related cytogenetic changes (AML-MRC) [2–8]. Morphological evidence of dysplasia alone is no longer a criterion for the diagnosis of AML-MRC in the most recent (2022) classifications of AML provided by the World Health Organisation (WHO), The International Consensus Classification of AML (ICC) and the European LeukemiaNet [6–8]. The pre-treatment karyotype is also used to assign patients to risk groups in order to determine whether standard therapies or more intensive forms of treatment are likely to be most effective [1, 9]. The presence of multiple abnormalities signifies that there is disease progression [1]. Chromosomal abnormalities have been described in over half of AML in adults but the frequency of specific cytogenetic abnormalities varies in different parts of the world [1, 10, 11]. It is also well-documented that the median age of AML patients in Western countries and Japan is about two decades higher than in the rest of the world [9–31]. Whether this is related to different pathogenic processes or is a reflection of the younger population profile is unclear. Reports of cytogenetic changes in AML from South Asian countries are limited, because a large proportion of patients do not have access to diagnostic technologies other than morphology [29–32]. We describe the chromosomal abnormalities seen in a large group of adult patients with AML diagnosed consecutively at our centre over 15 years and compare our findings with the literature.

## Patients and methods

### Patients

Karyotypes of all patients with AML aged ≥ 18 years seen at the Christian Medical College, Vellore between 2003 and 2017 and who underwent cytogenetic analysis at diagnosis were included in the analysis. Patients who had received chemotherapy and those with normal karyotypes with < 15 analysable metaphases were excluded.

### Cytogenetic analysis

Conventional cytogenetic analysis was performed on unstimulated overnight (or 48-h) cultures of bone marrow using standard protocols, and results reported as per the International System for Human Cytogenomic Nomenclature (ISCN) [33, 34]. Fluorescence in-situ hybridization (FISH) analysis was performed if the bone marrow morphology suggested that a specific abnormality could be present, to confirm a suspected abnormality if the chromosome morphology was suboptimal or to establish base-line values for follow-up post treatment.

### Descriptions of abnormalities

We used the terminology and definitions that most resembled the WHO 2016 and earlier classifications of AML for ease of comparison with previous studies because of slight differences between the 2022 WHO, ICC and ELN classifications of AML with respect to the definitions of MRC and complex karyotypes (CK) and the terminology used to describe the subtypes [6–8]. Therefore, we used the WHO/ICC definitions to describe complex karyotypes (≥ 3 abnormalities in the absence of class-defining RGA) unlike the ELN definition which also excluded hyperdiploid karyotypes without structural abnormalities (≥ 3 trisomies/polysomies only). Even though trisomy 8, monosomy 17 and the del 20q were termed MRC only by the ICC/ELN and the del 11q and del 13q/monosomy 13, only by the WHO, we categorised all these abnormalities as MRC. Monosomal karyotypes (MK) were those with two or more autosomal monosomies, or one single autosomal monosomy in addition to one or more structural chromosome abnormalities other than core-binding factor AML[8]. Apart from numerical abnormalities, balanced translocations (t) and unbalanced structural rearrangements were regarded as single abnormalities. Each abnormality in a karyotype was recorded separately to determine its absolute frequency and categorised as RGA, MRC or other. The karyotypes were also categorised hierarchically as described by Moorman et al., with each being assigned to only one of four mutually exclusive groups in the following sequence: translocations, inversions and insertions; deletions and monosomies; trisomies and duplications; normal karyotypes [35].

### Statistical analysis

Statistical analysis was performed using STATA 16 (Statcorp). One-way ANOVA was used to compare age differences between groups. We compared our findings with the West (Europe, U.K, USA and Australia), South-East (S.E) Asia (China, Hong Kong, Singapore, Malaysia, South Korea and Japan) and North (N.) Africa (Tunisia, Morocco and Egypt). Weighted average percentages of each abnormality were determined for all three regions (upto 18,850, 8971 and 1646 patients from the West, S.E Asia and N. Africa respectively) and the frequencies compared with our study using the one-sample proportion test. The value *p* < 0.05 was considered to be significant.

## Results

### ***Overview of patient characteristics and cytogenetic abnormalities (***Table 1***)***

**Table 1:** Overview of 1791 adult patients with AML

Characteristic	Total	Age	Males (%)	Females (%)
All patients	1791	42 (18–85)	1085 (60.6)	706 (39.4)
*Karyotype details*				
Normal karyotype*	646 (36.1)	45 (18–85)	398 (36.7)	248 (35.1)
Abnormal karyotype	1145 (63.9)	40 (18–82)	687 (63.3)	458 (64.9)
Single abnormality	567 (31.7)	39 (18–76)	332 (30.6)	235 (33.3)
Two abnormalities	299 (16.7)	38 (18–75)	185 (17.1)	114 (16.1)
≥ 3 abnormalities	279 (15.6)	43 (18–82)	170 (9.5)	109 (6.1)
Complex karyotype (WHO/ICC)**	200 (11.2)	48 (18–82)	131 (7.3)	69 (3.9)
Complex karyotype (ELN)***	184 (10.3)	42 (21–82)	126 (7)	58 (3.2)
Monosomal karyotype	158 (8.8)	48.5 (21–82)	110 (6.1)	48 (2.7)
*Types of abnormalities*				
RGA	585 (32.7)	35 (18–72)	330 (18.4)	255 (14.2)
MRC	403 (22.5)	46 (18–82)	262 (14.6)	141 (7.9)
Other^ abnormalities	157 (8.8)	42 (18–74)	95 (5.3)	62 (3.5)
AML-NOS (other^, and normal*)	803 (44.8)	44 (18–85)	493 (27.5)	310 (17.3)
*Moorman classification*				
Translocations	606 (33.8)	36 (18–76)	341 (19)	265 (14.8)
Deletions	323 (18)	46.5 (18–85)	217 (12.1)	106 (5.9)
Trisomies	216 (12.1)	43 (18–76)	129 (7.2)	87 (4.9)
*Blood counts*				
WBC count × 10^9^/L, n = 1759	7.1 (0.2–824)		7.1 (0.2–821.4)	7.1 (0.3–824)
Haemoglobin, g/dl, n = 1758	8.1 (2.1–18.6)		8.1 (2.3–18.6)	8 (2.1–15.6)
Platelet count × 10^9^/L, n = 1759	37 (2–1541)		38 (2–690)	36 (3–1541)

^*^ to indicate that normal karyotypes comprise AML-NOS; **WHO/ICC definition: ≥ 3 abnormalities in the absence of RGA ; ***, ELN definition: ≥ 3 abnormalities in the absence of RGA and hyperdiploid karyotypes without structural abnormalities; ^ , abnormalities other than RGA or MRC

There were 1860 patients with adult AML who presented at diagnosis, of whom 1791 (96.3%) fulfilled the criteria for inclusion. Patients ranged from 18–85 years (median 42 years); 1085 (60.6%) were males. Normal karyotypes were seen in 646 patients (36.1%) and were determined by analysis of ≥ 20 metaphases in 89% of patients and 15–19 metaphases in the remaining 11%. There were 1145 (63.9%) patients with abnormal karyotypes.

Solitary abnormalities were seen in 567 (31.7%) patients and two abnormalities in 299 (16.7%). There were 279 (15.6%) patients with ≥ 3 abnormalities including 79 with RGA; thus, there were 200 (11.2%) complex karyotypes as per the WHO/ICC classifications, and 184 (10.3%) as per the ELN definition, exclusive of 16 karyotypes with hyperdiploidy and no structural abnormalities. Monosomal karyotypes were seen in 158 (8.8%) patients, 123 (77.8%) of which were complex. Categorisation of abnormal karyotypes according to the 2022 WHO, ICC and ELN classifications showed RGA in 585 (32.7%) patients, MRC in 403 (22.5%), and abnormalities other than RGA or MRC (other abnormalities) in the remaining 157 (8.8%). Hierarchical (Moorman) classification of abnormal karyotypes showed 606 (33.8%) translocations, 323 (18%) deletions and 216 (12%) trisomies. These findings are shown in Table 1.

### ***Age distribution (***Table 1***, ***Figs. 1 and 2***)***

**Fig. 1:**
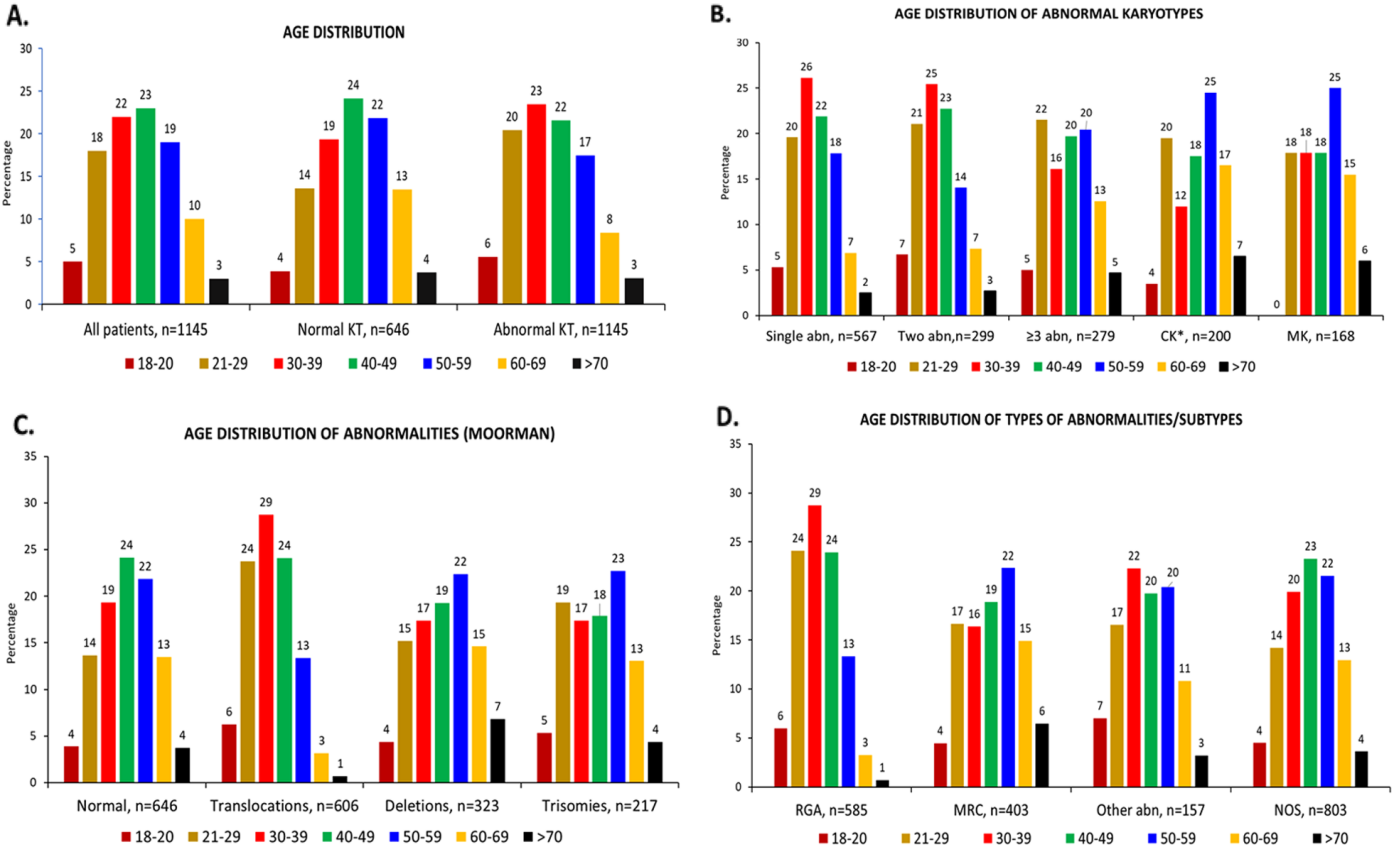
Age distribution: **A**. All patients, normal and abnormal karyotypes. **B**. One, two and three or more abnormalities, complex karyotypes and monosomal karyotypes. **C**. Abnormalities as per Moorman classification. **D**. Types of abnormalities/subtypes

**Fig. 2:**
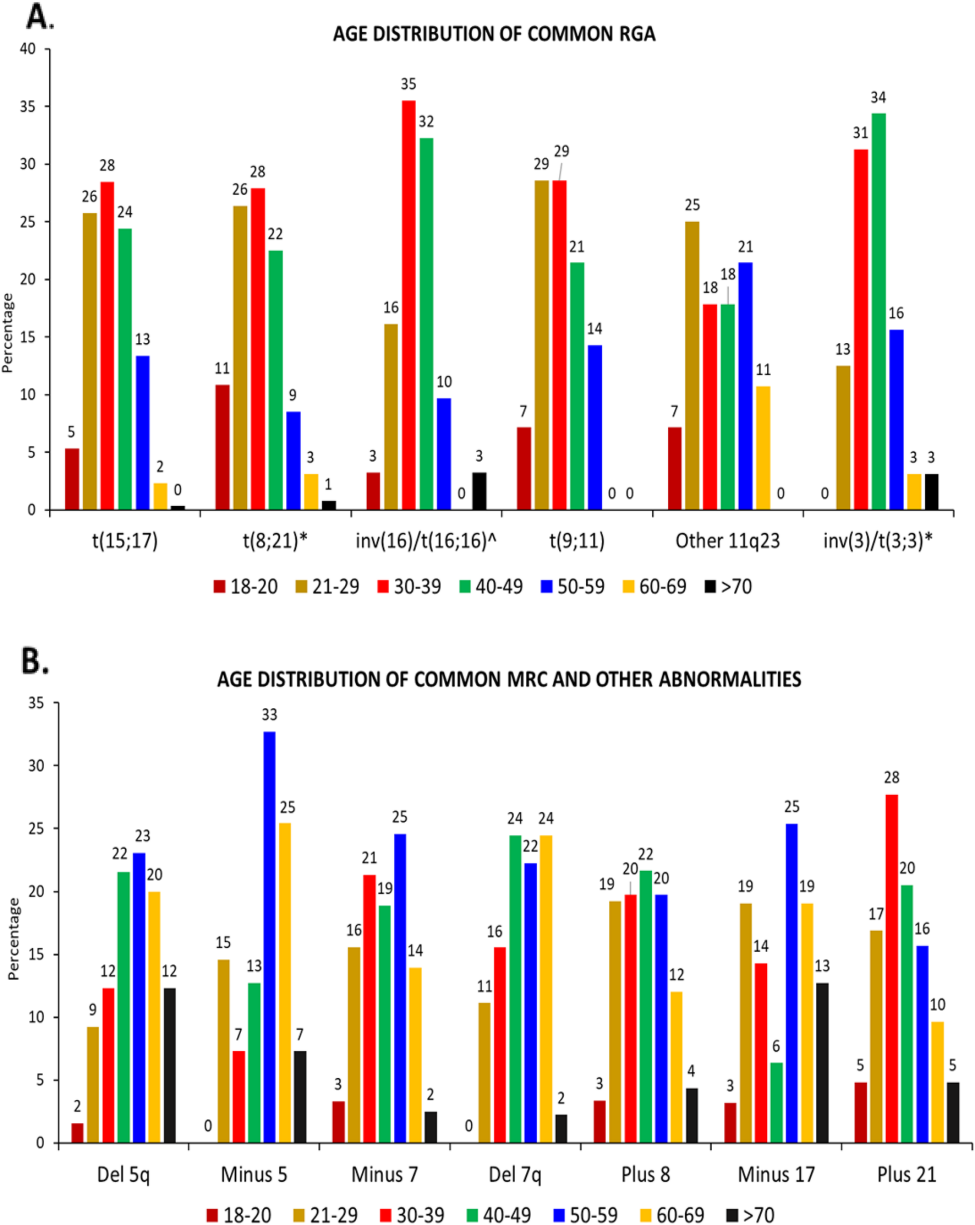
Age distribution of **A**. Common RGA. **B**. Common MRC and other abnormalities

Patients with normal karyotypes had a higher median age than those with abnormal karyotypes (45 vs 40 years, *p* < 0.001). Patients with ≥ 3 abnormalities also had a higher median age (43 years) than those with one or two abnormalities (39 years, *p* = 0.005) (Additional file 1). Patients with CK (WHO/ICC) had the highest median age (48 years, *p* < 0.001).

The number of patients progressively increased upto the fifth decade (68%) and declined subsequently (13% above 60 years) (Fig. 1A). The age distributions of normal and abnormal karyotypes were similar to the overall distribution, even when the latter was categorised into subgroups. However, there were differences in the age at which each category was most common. Normal karyotypes peaked a decade later (40–59 years) than abnormal karyotypes (Fig. 1A). Karyotypes with ≥ 3 abnormalities had almost the same frequency at 21–29 years (22%) and 40–59 years (20% each) (Fig. 1.B). Complex karyotypes and monosomal karyotypes (25% each), deletions (22%) and trisomies (23%) were most common at 50–59 years of age, two decades later than those with one or two abnormalities or translocations (Fig. 1.B & 1.C). The RGA were most common (29%) at 30–39 years of age, two decades earlier than MRC (22% at 50–59 years). Other abnormalities were also most common (22%) at 30–39 years; however, AML-NOS comprising other abnormalities and normal karyotypes was most common at 40–59 years (Fig. 1.D). The age distribution of the most common abnormalities is shown in Fig. 2.

### ***Cytogenetic subtypes (***Tables 2, 3, 4***)***

**Table 2:** Recurrent genetic abnormalities in AML

Abnormality	t(15;17)	t(8;21)^#^	inv(16)*	t(9;11)	inv(3)/t (3;3)^	t(6;9)	t(9;22)	Other t (v;11q23)^##^	t NUP98**	Rare t^^
*Patients, *n* (%)*										
All	299 (16.7)	129 (7.2)	31 (1.7)	14 (0.8)	32 (1.8)	16 (0.9)	19 (1.1)	28 (1.6)	8 (0.4)	10 (0.6)
M	162 (54.2)	77 (59.7)	13 (41.9)	9 (64.3)	23 (71.9)	12 (75)	11 (57.9)	14 (50)	3 (37.5)	4 (40)
F	137 (45.8)	52 (40.3)	18 (58.1)	5 (35.7)	9 (28.1)	4 (25)	8 (42.1)	14 (50)	5 (62.5)	6 (60)
Age	35 (18–75)	33 (18–76)	36 (20–71)	33 (18–58)	41 (24–73)	40.5 (21–65)	39 (23–57)	40.5 (18–65)	43 (22–67)	31 (20–54)
*P* value for age	Reference	0.04	0.53	0.52	0.02	0.06	0.26	0.22	0.14	0.12
*Karyotype complexity*										
Single abn	210 (70.2)	20 (15.5)	20 (64.5)	6 (42.9)	11 (34.4)	13 (81.3)	7 (36.8)	19 (67.9)	6 (75)	4 (40)
Two abn	60 (20.1)	88 (68.2)	5 (16.1)	5 (35.7)	13 (40.6)	3 (18.8)	7 (36.8)	6 (21.4)	1 (12.5)	3 (30)
≥ 3 abn	29 (9.7)	21 (16.3)	6 (19.4)	3 (21.4)	8 (25)	0	5 (26.3)	3 (10.7)	1 (12.5)	3 (30)
*Additional cytogenetic abnormalities*										
del 5q	1	1	–	–	4	–	–	1 t(11;19) q13.3)	–	–
Minus 7	–	3	–	1	15	–	3	–	–	–
del 7q	4	2	1	–	–	–	1	–	–	–
Plus 8	28	4	7	5	1	–	2	4 t(6;11)	1 t(7;11)	1 t (10;11)
del 11q	1v	–	–	–	–	–	–	–	–	–
Del/other 12p	–,1	–	–	–	–	–	–	–	–	1 t (10;11),–
Minus 13/del 13q	1,–	1,–	–	–	–/1	–	1,–	–	–	–
Minus 17	2	1	–	–	1	–	1	–	–	–
Iso/ider 17q***	2,7	–	–	–	–	–	–	–	1 t(7;11),–	–
Del/other 17p	–,1	–,1	–	–	–	–,1	–	–	–	–
Plus 4	2	5	–	–	1	–	–	–	–	–
Minus 5	–	1	–	–	1	–	–	–	–	–
del 9q	6	16	1	–	–	–	–	1	–	–
Minus 18	1	1	–	–	2	–	–	–	–	–
Plus 19	1	1	–	3	–	–	1	2 t(6;11)	1 t(7;11)	–
Plus 21	9	–	2	1	–	–	1	2 t(6;11)	–	–
Plus 22	1	–	6	1	1	–	–	–	–	–
Minus X	–	20	–	–	1	–	–	–	–	2 t(10;11),
Minus Y	–	54	–	–	–	1	–	–	–	1 t(16;21)

t, translocation; t(15;17)(q24;q21); ^#^t(8;21)(q22;q22) includes one with concurrent t(3;3); inv, inversion; *inv(16)(p13.1q22),n =30 and t (16;16)(p13.1;q22),n =1; t(9;11)(p13;q23); ^inv(3)(q21q26), n =17/t(3;3)(q21;q26),n =15; t(6;9)(p22;q34); t(9;22)(q34;q11.2); ^##^ Other t(v;11q23): t(6;11)(q27;q23.3),n =9; t (10;11)(p12.3;q23.3),n =5; t(10;11)(q21.3;q23.3),n =1; t(11;19)(q23.3;p13.1), n =4; t(11;19)(q23.3;p13.3),n =3; t(11;17)(q23;q25),n =3; t(X;11) (q22;q23),n =2; t(1;11)(q21;q23),n =1;**NUP98 translocations: t(7;11) (p15;p15),n =4; t(11;20)(p15;q12),n = 2; t(2;11)(q31;p15),n =1; t(11;12)(p15;p13),n = 1; ^^rare translocations: t(10;11)(p11;q14), n = 6; t (1;3)(p36;q21),t(3;5)(q25;q35),t(8;16)(p11;p13) & t(16;21)(p11.2;q22),n = 1 each; abn,abnormality; del,deletion; ***,iso/ider17q, isochromosome 17q and isoderivative 17q.

**Table 3:** Myelodysplasia-related cytogenetic abnormalities

Abnormality	**Plus 8**	**Del 5q**/**add/t 5q**	**Minus 7**	**Del 7q/t 7q**	**Del 11q**	**Del 12p/add/t 12p**	**Minus 13/del 13q**	**Minus 17**	**Del 17p/add**/**t 17p**	**i 17q**	**Del 20q**
Patient details, n (%)`											
All	208 (11.6)	65 (3.6)/4 (0.2)/1 (0.06)	122 (6.8)	45 (2.5)/5 (0.3)	14 (0.8)	15 (0.8)/16 (0.9)/3 (0.2)	26 (1.5)/9 (0.5)	63 (3.5)	10 (0.6)/17 (0.9)/4 (0.2	11 (0.6)/	11 (0.6)
M	121 (58)	41 (63)/3 (75)/0	82 (67)	31 (69)/2 (40)	9 (64)	10 (67)/11 (69)/2 (67)	19 (73)/4 (44)	47 (75)	7 (70)/9 (53)/4 (100)	6 (55)/	10 (91)
F	87 (42)	24 (37)/1 (25)/1 (100)	40 (33)	14 (31)/3 (60)	5 (36)	5 (33)/5 (31)/1 (33)	7 (27)/5 (56)	16 (25)	3 (30)/8 (47)/0	5 (45)/	1 (9)
Age	43 (18–76)	52 (19–82)/46 (25–61)/26	45 (18–74)	49 (21–72)/52 (31–55)	38 (19–78)	40 (19–57)/55 (26–78)/42 (20–72)	48 (21–82)/35 (19–63)	53 (18–82)	51 (32–70)/55 (20–73)/59.5 (55–70)	45 (18–65)	58 (25–77)
*P* value, age	Reference	0.002/0.82/0.38	0.29	0.04/0.75	0.51	0.1/0.41/0.15	0.45/0.41	0.01	0.08/0.09/0.02	0.85	0.12
Karyotype complexity											
Single abn	67 (32.2)	10 (15.4)/0/0	30 (24.6)	11 (24.4)/0	0	2 (13.3)/0/1 (33.3)	0/0	3 (4.8)	4 (40)/0/1 (25)	1 (9.1)	1 (9.1)
Two abn	50 (24)	11 (6.9)/0/0	35 (28.7)	12 (26.7)/1 (20)	3 (21.4)	7 (46.7)/3 (18.8)/1 (33.3)	0/1 (11.1)	4 (6.3)	0/2 (11.8)/1 (25)	3 (27.3)/	6 (54.5)
≥ 3 abn	91 (43.8)	44 (67.7)/2 (50)/1 (100)	57 (46.7)	22 (48.9)/4 (80)	11 (78.6)	6 (40)/13 (81.3)/1 (33.3)	26 (100)/8 (88.9)	56 (89)	6 (60)/15 (88.2)/2 (50)	7 (63.6)	4 (36.4)
Association with RGA											
Double	23 (11.1)	2 (3.1)/1 (25)/0	14 (11.5)	4 (8.9)	0	1 (6.7)/1 (6.3)/0	0/0	0	0/1 (5.9)/0	2 (18.2)	–
≥ 3	29 (13.9)	5 (7.7)/0/1 (100)	8 (6.6)	4 (8.9)/0	1 (7.1)	0/0/0	3 (11.5)/1 (11)	5 (7.9)	0/2 (11.8)/0	1 (9.1)	–
Plus 4	20	5/–/–	6	2/–	1	–	4/–	3	–/2/–	1	–
Minus 5	17	9/–/–	25	8/–	3	4/8/–	12/3	26	3/7/1	1	–
del 5q	12	–/–/–	15	5/1	2	1/5/1	5/3	13	2/6	2	–
add 5q	–	–	1	1/–	–	–	–	2	–	–	–
t 5q	1	–	–	–	–	–	–	–	–	–	–
Minus 7	18	15/1/–	–	6/–	2	3/4/1	14/–	16	3/2/	2	–
del 7q	10	5/1/–	6	–/–	3	–/3/–	3/–	7	–/2	1	–
t 7q	2	1/–/–	–	–	–	–	–	–	–/1/–	–	–
Plus 8	–	12/–/1	18	10/2	2	4/2/–	10/–	13	4/4	2	–
del 9q	6	6/–/–	4	5/–	1	2/1/–	–	3	1/–/–	–	–
del 11q	2	3/–/–	2	3/–	–	–/2/–	1/1	5	1/–	1	–
del 12p	4	1/–/–	3	–/–	–	–/2/–	1/–	–	1/–/–	–	–
add 12p	2	5/–/–	4	1/–	2	2/–/–	–/1	4	1/2/–	–	–
t 12p	–	1/–/–	1	1/–	–	–/–/–	–/–	–	–/1/–	–	–
Minus 13	10	5/–/–	14	3/–	1	1/–/–	–/–	1	1/1/–	–	–
del 13q	–	3/–/–	–	–/–	1	–/1/–	–/–	1	1/2/–	–	–
Minus 17	13	13/2/–	16	7/–	5	–/4/–	15/2	–	3/2/–	1	–
del 17p	4	2/–/–	3	–/–	1	1/1/–	1/1	3	–/–/–	–	–
add 17p	3	6/–/–	2	2/1	–	–/2/1	1/2	2	–/–/–	1	–
t 17p	1	1/–/–	1	1/–	–	–/–/–	–/–	–	–/–/–	–	–
i 17q	2	1/–/–	2	1/–	1	–	–/1	1	–/1/–	–	–
Minus 18	17	10/–/–	17	4/–	3	2/3/–	14/2	26	5/–	–	–
Plus 19	20	5/–/–	8	1/–	1	–/–/–	5/1	9	3/2/–	–	–
Plus 21	36	6/–/–	14	4/1	2	–/1/–	4/–	12	5/–	1	–
Plus 22	24	6/–/–	5	3/–	3	1/2/–	3/–	6	5/–	1	–
Minus X	10	1/–/1	5	1/–	2	1/1/–	4/–	4	1/3/–	–	–
Minus Y	3	4/–/1	5	1/–	1	–/1/–	3/–	8	2/1/–	–	–

Abn: abnormality/abnormalities; del, deletion; add, addition; t, translocation; minus, monosomy; *iso, isochromosome

**Table 4:** Abnormalities other than RGA and MRC (n =  ≥ 20)

Abnormality	Patient characteristics, n (%)					Age distribution, years, n (%)				Karyotype complexity, n (%)			Assoc with RGA	
	Total	M	F	Age	*p*	18–20	21–39	40–59	≥ 60	Single	Two	≥ 3	Double	≥ 3
Minus 5	55 (3.1)	37 (67.2)	18 (32.8)	54 (21–78)	Ref*	0	12 (21.8)	25 (45.5)	18 (32.7)	0	0	55 (100)	0	2 (5.5)
Plus 4	54 (3)	35 (64.8)	19 (35.2)	38 (18–73)	0.001	5 (9.3)	23 (42.6)	18 (33.3)	8 (14.8)	10 (18.5)	14 (25.9)	30 (55.6)	4 (7.4)	4 (7.4)
Plus 6	33 (1.8)	22 (66.7)	11 (33.3)	43 (18–65)	0.009	2 (6.1)	13 (39.4)	14 (42.4)	4 (12.1)	2 (6.1)	1 (3)	30 (90.9)	0 (0)	2 (6.1)
del 6q	20 (1.1)	15 (75)	5 (25)	40.5 (18–78)	0.02	1 (5)	9 (45)	7 (35)	3 (15)	0	2 (10)	18 (90)	1 (5)	1 (5)
Plus 9	28 (1.6)	17 (60.7)	11 (39.3)	39 (18–73)	0.01	2 (7.1)	13 (46.4)	8 (28.6)	5 (17.9)	4 (14.3)	3 (10.7)	21 (75)	0 (0)	1 (3.6)
Minus 9	26 (1.5)	18 (69.2)	8 (30.8)	36 (21–78)	0.07	0 (0)	14 (53.8)	6 (23.1)	6 (23.1)	0 (0)	2 (7.7)	24 (92.3)	0 (0)	2 (7.7)
del 9q	60 (3.4)	36 (60)	24 (40)	37 (18–78)	< 0.001	4 (6.7)	28 (46.7)	20 (33.3)	8 (13.3)	14 (23.3)	12 (20)	34 (56.7)	9 (15)	15 (25)
Plus 10	26 (1.5)	17 (65.4)	9 (34.6)	45.5 (20–78)	0.06	1 (3.8)	10 (38.5)	8 (30.8)	7 (26.9)	1 (3.8)	2 (7.7)	23 (88.5)	0 (0)	2 (7.7)
Minus 10	20 (1.1)	13 (65)	7 (35)	46 (21–75)	0.05	0 (0)	7 (35)	10 (50)	3 (15)	0 (0)	1 (5)	19 (95)	0 (0)	2 (10)
Plus 11	36 (2)	23 (63.9)	13 (36.1)	53 (19–78)	0.21	2 (5.6)	9 (25)	17 (47.2)	8 (22.2)	9 (25)	6 (16.7)	21 (58.3)	1 (2.8)	1 (2.8)
Minus 11	22 (1.2)	12 (54.5)	10 (45.5)	50.5 (20–78)	0.24	1 (4.5)	7 (31.8)	7 (31.8)	7 (31.8)	0 (0)	0 (0)	22 (100)	0 (0)	2 (9.1)
Minus 12	34 (1.9)	24 (70.6)	10 (29.4)	53 (21–77)	0.21	0 (0)	9 (26.5)	18 (52.9)	7 (20.6)	0 (0)	0 (0)	34 (100)	0 (0)	3 (8.8)
Plus 13	41 (2.3)	28 (68.3)	13 (31.7)	53 (18–75)	0.14	2 (4.9)	11 (26.8)	18 (43.9)	10 (24.4)	5 (12.2)	4 (9.8)	32 (78)	0 (0)	4 (9.8)
Plus 14	27 (1.5)	18 (66.7)	9 (33.3)	43 (18–73)	0.05	1 (3.7)	10 (37)	11 (40.7)	5 (18.5)	0 (0)	2 (7.4)	25 (92.6)	1 (3.7)	1 (3.7)
Minus 14	20 (1.1)	16 (80)	4 (20)	39 (24–59)	0.09	0 (0)	5 (25)	12 (60)	3 (15)	0 (0)	0 (0)	20 (100)	0 (0)	3 (15)
Minus 16	38 (2.1)	25 (65.8)	13 (34.2)	53 (20–82)	0.44	1 (2.6)	14 (36.8)	9 (23.7)	14 (36.8)	0 (0)	0 (0)	38 (100)	0 (0)	1 (2.6)
Minus 18	48 (2.7)	34 (70.8)	14 (29.2)	49.5 (18–78)	0.09	1 (2.1)	15 (31.3)	18 (37.5)	14 (29.2)	0 (0)	3 (6.3)	45 (93.8)	1 (2.1)	3 (6.3)
Plus 19	45 (2.5)	34 (75.6)	11 (24.4)	40 (18–73)	0.004	2 (4.4)	20 (44.4)	14 (31.1)	9 (20)	5 (11.1)	2 (4.4)	38 (84.4)	1 (2.2)	8 (17.8)
Minus 20	24 (1.3)	18 (75)	6 (25)	53 (19–78)	0.77	1 (4.2)	7 (29.2)	9 (37.5)	7 (29.2)	0 (0)	2 (8.3)	22 (91.7)	0 (0)	2 (8.3)
Plus 21	83 (4.6)	46 (55.4)	37 (44.6)	40 (18–73)	< 0.001	4 (4.8)	37 (44.6)	30 (36.1)	12 (14.5)	16 (19.3)	10 (12)	57 (68.7)	1 (1.2)	14 (16.9)
Minus 21	24 (1.3)	16 (66.7)	8 (33.3)	48.5 (21–78)	0.1	0 (0)	11 (45.8)	7 (29.2)	7 (29.2)	0 (0)	0 (0)	24 (100)	0 (0)	0 (0)
Plus 22	43 (2.4)	30 (69.8)	13 (30.2)	43 (18–78)	0.003	2 (4.7)	18 (41.9)	16 (37.2)	7 (16.3)	1 (2.3)	7 (16.3)	35 (81.4)	3 (7)	6 (14)
Minus 22	22 (1.2)	14 (63.6)	8 (36.4)	53 (20–78)	0.28	1 (4.5)	6 (27.3)	9 (40.9)	6 (27.3)	0 (0)	0 (0)	22 (100)	0 (0)	2 (9.1)
Minus X	37 (2.1)	7 (18.9)	30 (81.1)	37 (18–70)	< 0.001	4 (10.8)	18 (48.6)	12 (32.4)	3 (8.1)	0 (0)	16 (43.2)	21 (56.8)	14 (37.8)	9 (24.3)
Minus Y	81 (4.5)	0 (0)	81 (100)	36 (18–74)	< 0.001	7 (8.6)	39 (48.1)	25 (30.9)	10 (12.3)	10 (12.3)	47 (58)	24 (29.6)	47 (58)	10 (12.3)

^*^Reference for *p* value

The t(15;17) was our most common abnormality (16.7%), followed by trisomy 8 (11.6%), monosomy 7/del 7q (9.3%), the t(8;21) (7.2%), monosomy 5/del 5q (6.7%) and monosomy 17/del 17p (5.2%).

### ***Recurrent Genetic Abnormalities (RGA***) (Table 2***)***

The t(15;17) accounted for over half (51%) of the 585 RGA. An isoderivative 17q resulting in loss of 17p was present in seven patients (2.3%). There were three (1%) variant translocations: a t(11;17)(q23;q21), an unbalanced t(5;17)(q35;q21) and a three-way translocation involving chromosome 5q13. The t(8;21) was the next most common RGA (22%) RGA, with four (3.1%) variant translocations comprising three three-way translocations involving chromosomes 3q21, 6p23 and 12q15 and a four-way translocation involving chromosomes 1q22 and 13q34.

The inv(16)(p13.1q22)/t(16;16)(p13.1q22) and the inv(3)(q21q26)/t (3;3) (q21;q26) had similar frequencies (5.3% and 5.5% of RGA respectively). Translocations of chromosome 11q23 (KMT2A/MLL) accounted for 7% of all RGA, and the remaining RGA for ≤ 3% each. The inv(16), NUP98 translocations and the rare RGA were more common in females (M:F ratios 1:1.4, 1:1.7 and 1:1.5 respectively) although the latter two were few in number; other 11q23 translocations were equally common in males and females (Additional file 2). Additional cytogenetic abnormalities (ACA) were seen in almost half (46.2%) of all RGA, comprising the majority (57–84.5%) of the t(8;21), inv(3) /t(3;3), t(9;22) and t(9;11). The most common associations were: loss of a sex chromosome (57.4%) with the t(8;21), monosomy 7 (46.9%) with the inv(3)/t(3;3), trisomy 8 (35.7%) with the t(9;11) and trisomy 8 (22.6%) and trisomy 22 (19.4%) with the inv(16)/t(16;16). Trisomy 8 (9.4%) was also the most common ACA associated with the t(15;17) which was usually solitary (70%). Compared to the t(15;17), the median age of the t(8;21) was slightly lower (33 vs 35 years, *p* = 0.04), that of the inv(3)/t(3;3) was higher (41 vs 35 years, *p* = 0.02) while the other RGA were comparable. These findings are shown in Table 2.

### ***Myelodysplasia-related cytogenetic abnormalities (MRC) (***Table 3***)***

There were 403 (22.5%) patients with MRC as defined by the WHO and the ICC/ELN classifications. The most common MRC were trisomy 8 (11.6%), monosomy 7 (6.8%)/del 7q (2.5%), del 5q (3.6%) and monosomy 17 (3.5%). The idic(X)(q13) was not present. The del 13q and t 7q were slightly more common in women (M:F ratio 1:1.3–1.5). The majority (67.8–100%) of each MRC had ACA. The most frequent associations were: trisomies 8 and 21 (17.3%), monosomies 7 and 5 (20.5%), monosomy 7/del 7q and monosomy 5/del 5q (31.7%), and monosomy 17 with monosomies 5 and 18 (41.3% each). RGA were associated with 7–25% of each MRC except the del 20q. Monosomy 13 (100%) and the majority of monosomy 17, del 13q (89% each), del 11q (78.6%) and del 5q (67.7%) and almost half 44–49%) of the other MRCs were part of karyotypes with ≥ 3 abnormalities. The median age of patients with trisomy 8 (43 years) was lower than those with most of the other MRC (49–59.5 years, *p* =  < 0.05). Monosomy 7/del 7q and monosomy 5/del 5q were seen concurrently in 53 (13.2%) patients. These findings are summarised in Table 2.

### ***Abnormalities other than RGA and MRC (***Table 4***):***

There were 157 (8.8%) patients with these abnormalities, the most common being trisomy 21 (4.6%) and minus Y (4.5%). Monosomies 5, 11, 12, 14, 16, 21 and 22 were always present in karyotypes with ≥ 3 abnormalities. The majority (upto ~ 95%) of each of the other abnormalities in this group were also part of such karyotypes except for minus Y, minus X, del 9q and trisomy 4 which were often associated with RGA. The other trisomies reported in AML (trisomies 4, 6, 11, 13,14 and 19) were seen in 2–3% of patients. Monosomy 5 was associated with a significantly higher median age (53 vs.36–43 years, *p* < 0.001 to *p* = 0.02) than several others in this group (trisomies 4, 6, 9,19,21 and 22, del 6q, del 9q, minus X and minus Y). These findings are summarised in Table 4. There were 40 (2.2%) balanced translocations other than AML-RGA including four which were previously (WHO 2016) termed myelodysplasia-related, namely, the t(1;16)(p31;q24), t(1;21)(p36;q22), t(4;12)(q12;p13) and t(5;12)(q32;p13.2). The remaining 36 (2.1%) were novel translocations of which 16 involved 7q, 5q, and 3q (four each), 12p13 (three) and 21q22 (one), including one t 7q17p and one t 3q12p each (Additional file 3).

### Complex karyotypes

The 200 CK included 109 (54.5%) with abnormalities of chromosomes 5 and/or 7.

Monosomy 5 or del 5q was present in 84 (46.5%) CK and monosomy 7 or del 7q in 62 (33.5%); these abnormalities were concurrent in 45 (22.5%) CK. CK with abnormalities of chromosomes 5 and 7 had a greater number of aberrations (3–28, median 10) than those without these abnormalities (3–23, median 4). These patients also had a higher median age (53 years, range 18–82 years) than those without these abnormalities (40 years, range 18–72 years). Other abnormalities frequently associated with CK were monosomies 17 and 18 in 51 (25.5%) and 42 (21%) karyotypes respectively and trisomies 8 and 21 in 61 (30.5%) and 43 (21.5%) karyotypes respectively. (Additional file 4).

### Monosomal karyotypes

There were 158 (8.8%) MK comprising 135 (7.5%) with ≥ 3 abnormalities and 23 (1.5%) with two abnormalities; 123 (77.8%) karyotypes were complex.. The MK included 23 (14.5%) AML-RGA which were distributed as follows: inv(3), n = 11; t(3;3), n = 5; t (9;22), n = 6 and t(9;11), n = 1. Twelve of the AML-RGA had ≥ 3 abnormalities. Monosomy 7 was seen in 72 (45.6%). MK, with 17 (43.5%) associated with RGA. Monosomies 5, 17, 18, 16 and 12 were the other common monosomies seen in 51 (32.3%), 49 (31%), 42 (26.6%), 34 (21.5%) and 30 (19%) MK respectively. Monosomy 5 was associated with one or more additional monosomies in all but one of these karyotypes. Monosomies 5 and 7 were seen concurrently in 22 (13.9%) MK.

### *Categorisation into cytogenetic risk groups*:

There were 459 (25.6%) patients whose karyotypes were in the favourable risk group and 374 (21%) in the unfavourable risk group which comprised 96 (5.4%) RGA, 78 (4.4%) high-risk MRC, namely, del 5q/monosomy 5, monosomy 7, monosomy 17/abn 17p and 200 (11.2%) CK. The 958 (53.5%) patients in the intermediate risk group consisted of 312 (17.4%) patients with abnormal karyotypes and 646 (36.1%) with normal karyotypes. The abnormal karyotypes in the intermediate risk group comprised 31 (2%) with RGA, 121 with MRC (6.8%) and 160 (15.7%) with other abnormalities (Additional file 5).

## Discussion

We have described the demographic and cytogenetic profile of a large series of consecutively evaluated adult patients with AML presenting to a tertiary care centre for haematological disorders in southern India and compared our findings with the West, S.E.Asia and N.Africa (upto 18,850, 8971 and 1646 patients respectively as shown in Tables 5, 6, 7 and Additional files 6–8) [9–28]. These reports varied with respect to the criteria used for inclusion as shown in Tables 5, 6, 7. Some studies categorised patients hierarchically [9, 11, 13, 16, 22, 25]. Others did not include one or more of the following major abnormalities: the t(15;17), the inv(16) and trisomy 8 [9, 11, 12, 15, 19]. However, the age at presentation and patterns of cytogenetic abnormalities were fairly consistent in each geographic region, barring one or two studies in which some frequencies differed from others in the same region [15, 20, 24, 27, 28].

**Table 5:** Comparison of findings with Western literature

Country	India	Germany	USA	UK	UK	Spain	Sweden	Germany	Australia	Australia
Reference	This study	Bacher	Byrd	Sanderson	Grimwade	Sierra	Lazarevic	Creutzig	Nakase	Gangatharan
Year	2023	2005	2002	2006	2010	2006	2014	2016	2000	2021
Duration of study	2003–17	1996–04	1984–95	1983;98–06	1988–09	1995–02	1997–06	1998;05–12	1986–97	2009–18
Type of study	Hosp	Hosp	CALGB	Pop	MRC	Hosp	Pop	Hosp	Hosp	Hosp
No. of cases	1791	2460	1311	1709	5876	1271	3251	4516	368	734
Karyotypes analysed	1791	2235	1213	1192	5876	1129	1893	4372	230	710
Age, median (range)	42 (18–85)	NA (16–90)	52 (15–86)	62 (16–99)	44 (16–59)	61 (1–94)	66 (18- > 80)	0–100	51* (16–86)	64 (16–94)
Male:Female ratio	1.5:1	NA	1:1	1.1:1	NA	1.2:1	NA	NA	1.3:1	1.3:1
Normal	36.1	58	48	45	41	36	43	49	44	39
Abnormal	63.9	42	52	55	59	64	57	51	56	61
inv(3)/t(3;3)	1.8	1.6	1.0	NA	1	3.2` 3q	1	1.3	NA	0.7
Del (5q)/minus 5	3.6/3.1	1.7	3.5/2.1	5/2	2,2	9.1	13	1.5	13.5***	0.5
t (6;9)	0.9	NA	0.7	NA	1	NA	0.2	0.3	NA	NA
Minus 7/del (7q)	6.8/2.5	2.6	3.9/1.6	3/2	5,2	8.6	13	1.8	13.5***	3.5
Plus 8	11.6	5.7	10.1	6	10	11.4	4.3	5.6	13.5***	5.7
t(8;21)	7.2	4.3	6.7	4	7	2.7	1.9	2.6	5.2	3.1
t(9;22)	1.1	NA	NA	1	1	NA	NA	NA	NA	0.1
t(9;11)	0.8	NA	2.2	1	1	NA	0.7	0.8	NA	NA
Other/all^11q23	1.6	2.7^	2.3	1	3	3.3^	1.1	1.5	2.6^	3.5^
t(15;17)	16.7	NA	NA	8	13	14.8	NA	5.2	11.7	7.8
inv(16)/t(16;16)	1.7	NA	7.9	2	5	2.7	2.2	2.5	6.1	1.9
Minus 17/abn 17p	3.5/1.7	0.2 (i 17q)	5.1	NA	2,2	NA	8.8	0.4	NA	NA
Plus 21	4.6	0.8	2.3	NA	3	NA	NA	NA	NA	0.7
Complex( ≥ 3)/CK**	15.6/11.2**	12.3	11.1	15	< 14**	19	24	14.5	NA	20.6

UK, United Kingdom; USA, United States of America; Hosp, hospital-based; CALGB trial; MRC trial; Pop, population-based; *Mean age; **CK as per WHO & ICC 2022, exact number not specified; ***, combined frequency of chromosomes 5,7,8; ^, all 11q23 abnormalities

**Table 6:** Comparison of findings with reports from South-East Asia

Country	India	Japan	Japan	Singapore	China	China	Hong Kong	Korea	Malaysia	Malyasia
Reference	This study	Nakase	Wakui	Enjeti	Cheng	Li	So	Byun	Meng	Ambayya
Year	2023	2000	2008	2002	2009	2012	2011	2016	2013	2021
Duration	2003–17	1986–97	12/97–07/01	9/91–3/03	12/94–11/07	2003–10	89- 06/09	2007–11	2007–12	2012 -06/19
Type	Hosp	Hosp	AML-97^#^	Hosp	Hosp	Hosp	Hosp	Hosp	Hosp	Hosp
No. of cases	1791	494	809	501	1432	2516	834	2806	480	854
Karyotypes analysed	1791	436	638	454	1293	2308	629	2717	480	601
Age, median (range)	42 (18–85)	51* (16–91)	45 (15–66)	48 (15–100)	42 (4–84)	37 (0.2–86)	43 (0.3–70)	50 (14–88)	39 (0.3–81)	45 (12–93)
Male: Female ratio	1.5:1	1.4:1	1.6:1	1.4:1	1.2:1	1.3:1	1.2:1	1.2:1	1:1	1.1:1
Normal	36.1	43	42	39	42.3	39.8	39	42	70	49
Abnormal	63.9	57	58	61	57.7	60.2	61	58	30	51
inv(3)/t(3;3)	1.8	NA	0.8	0.7	NA	0.6	1	0.4	NA	NA
Del (5q)/minus 5	3.6/3.1	7.5***	0.3	6.6	0.8	0.3/1.4	4^^	3.4	0.8	5.3^^
t(6;9)	0.9	NA	0.6	0.7	NA	NA	< 1	0.8	NA	NA
Minus 7/del (7q)	6.8/2.5	7.5***	0.3	7	1.4	1.2/1.5	4^^	4.6	1.2	5.3^^
Plus 8	11.6	7.5***	NA	7.3	3.8	5.5	4	7.0	3	3.2
t(8;21)	7.2	13.2	17.7	7.5	8.3	15.1	9	9.7	7.5	8.5
t(9;22)	1.1	NA	1.1	NA	1.8	1.5	< 1	NA	NA	NA
t(9;11)	0.8	NA	1.6	0.9	NA	0.3	1	0.8	NA	NA
Other/all^11q23	1.6	2.3^	3.4	1.3	1.2^	1.3	< 1	1.8	< 2^	2.5^
t(15;17)	16.7	11.2	NA	11	14.3	16.7	16	10.7	2.3	14.3
inv(16)/t(16;16)	1.7	2.7	4.1	1.1	NA	2.1	3	4.2	< 2^##^	5
-17/abn 17p	3.5/1.7	NA	NA	NA	NA	1.0/0.8	NA	NA	NA	NA
Plus 21	4.6	NA	NA	NA	1.6	2.4	1^^^	NA	NA	NA
Complex (≥ 3)/CK**	15.6/11.2**	NA	6.4^##^	17	6.4	8.6	4^^^^	11.7	7.3**	NA

^#^,JALSG Trial; *,mean age; **,CK as per WHO & ICC 2022; ***,combined frequency of chromosomes 5,7,8; ^, all 11q23 abnormalities; ^^, combined frequency of chromosomes 5 and 7; ^^^,trisomy and tetrasomy 21; ^^^^,complex del (5q)/-7/del (7q) only; ^##^,CK ≥ 4

**Table 7:** Comparison of findings with reports from North Africa & South Asia

Country	India	Tunisia	Morocco	Egypt	Pakistan	India	India
Reference	This study	Gmidene	Khoubila	El-Naggar	Shaikh	Amare	Namratha
Year	2023	2012	2019	2021	2018	2016	2020
Duration of study	2003–17	2000–07	2004–14	2019–21	2011–16	2008–15	2013–14
Type of study	Hosp-based	Hosp based	Hosp-based	Hosp-based	Hosp-based	Hosp-based	Hosp-based
No. of cases	1791	631	927	120	321	2042	203
Karyotypes analysed	1791	631	895	120	288	1906	173
Age, median (range)	42 (18–85)	37 (8 dy–95y)	40.5 (20–60)	36.5 (18–86)	NA, ≥ 15	38 (16 -86)	39 (16–82)
Male:Female ratio	1.5:1	1.3:1	1.1:1	1.1:1	1.7:1	1.5:1	1.1:1
Normal karyotypes	36.1	37.1	42	56.7	61.1	NA	34.6
Abnormal karyotypes	63.9	62.9	58	43.3	38.9	NA	65.4
inv(3)/t(3;3)	1.8	NA	0.6	1.6	NA	1.5	3.4 all 3q
Del (5q)/minus 5	3.6/3.1	NA	0.5	nil	NA	3.4	2.3 del 5
t (6;9)	0.9	NA	NA	NA	0.7	NA	1.1
Minus 7/del (7q)	6.8/2.5	3	2.9/-	0.8	1	6.3	1.1 del 7
Plus 8	11.6	7	4.5	3.3	2.4	8.4	NA
t(8;21)	7.2	12.2	12.5	7.5	8.3	14.7	20.8
t(9;22)	1.1	NA	NA	0.8	NA	NA	NA
t(9;11)	0.8	NA	1	NA	NA	2	3.4
Other/all^11q23	1.6	3.8^	2.6	7.5^	NA	3	NA
t(15;17)	16.7	13.2	3.7	9.2	4.9	9.4	8.6
inv(16) /t(16;16)	1.7	3.5	3.3	7.5	**0.7**	4.5	21.3^^
Minus 17/abn 17p	3.5/1.7	NA	NA	0.8 iso 17q	NA	2	0.5
Plus 21	4.6	NA	NA	NA	NA	NA	NA
Complex (≥ 3)/CK**	15.6/11.2**	10.8	7.4	0.8	9	NA	2.3

^, all 11q23 abnormalities; ^^, inv(16),17.9%, t(16;16), 3.4%; **,CK as per WHO & ICC 2022

### ***Comparison of age distribution (***Tables 5, 6, 7***)***

The median age of our patients was lower than in the West (42 vs 52–66 years) as shown in Table 5 even when similar age groups were compared (39 vs 44 years in those ≤ 59 years as reported by Grimwade et al.) [9, 10, 13–15, 17, 18]. It was also lower than in one study from Japan (mean age 51.4 years) but comparable to another, as well as the rest of Asia and N. Africa (37–48 years) [11, 17, 19–28]. The lower age in most of Asia and N. Africa (Tables 6 and 7 respectively) could be due to geographic and/or ethnic differences in the response to environmental factors that predispose to the development of leukemia.

The decline in the number of patients after the sixth decade (13% ≥ 60 years) was similar to several reports from Asia (14–24% ≥ 60 years) but unlike Korea and the West (34–77% ≥ 60 years) [11, 15, 16, 18, 21–25, 35]. The age distribution of abnormalities was also approximately the same across each decade unlike the West in which deletions and trisomies increased with age (51% and 35% respectively in those ≥ 60 years) [12, 13, 15, 35]. The peak age of our normal karyotypes (40–49 years) was two decades lower than in the West (≥ 60 years); it differed from both Malaysian reports (50–59 years and ≤ 30 years) [12, 15, 16, 21, 24, 25, 35].

Our t(15;17), t(8;21) and inv(16), though seen in all age groups, were most common in patients below 40 years, similar to most reports from S.E.Asia [11, 21, 24, 25]. However, the t(15;17) and t(8;21) were equally common at 40–49 years (28% and 25% respectively) in one Chinese study each [11, 21]. In contrast, the peak frequencies of RGA varied from 20–29 years to 40–49 years in the West [15, 16, 35].

**Fig. 3:**
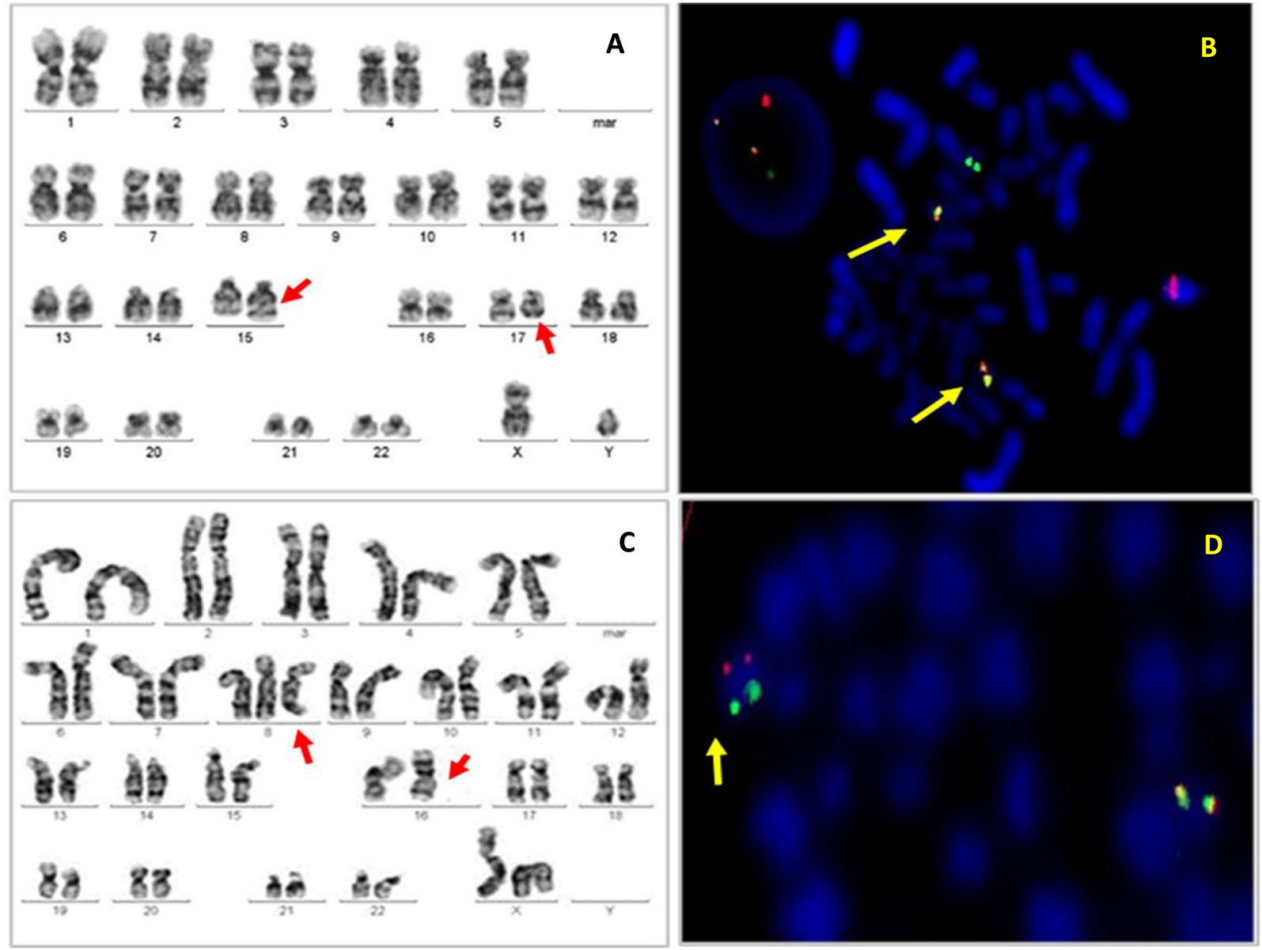
**A**. Karyotype: 46,XY,t(15;17)(q24;q21). **B**. FISH: dual colour, dual fusion probe for chromosome 15 (PML, red) and chromosome 17 (RARA, green). Arrows show fusion signals on both derivative chromosomes. **C**. Karyotype: 46,XX,+8, inv (16)(p13.1q22). **D**. FISH, dual colour break-apart rearrangement probe for chromosome 16q22.1 (CBFB). Fusion signal on normal chromosome 16 (red, 5’CBFB; green, 3’CBFB). Arrow shows the derivative chromosome 16 with separate red and green signals. Karyotypes are G-banded. FISH from Abbott Molecular, Des Plaines, IL, USA)

Trisomy 8 was most common in the age group 40–49 years and abnormalities of chromosomes 5 and 7 (high-risk abnormalities) at 50–59 years, one to two decades earlier than in the West (60- > 80 years) [16, 35]. The age distribution of these abnormalities which were relatively uncommon in most of S.E.Asia were similar to our study [11, 21, 25]. Deletions are thought to be more common in older individuals because they are considered to be a result of cumulative DNA damage [1, 35] (Fig. 3).

### ***Comparison of frequency of abnormalities (***Tables 5, 6,7*** & ***Fig. 4***)***

**Fig. 4:**
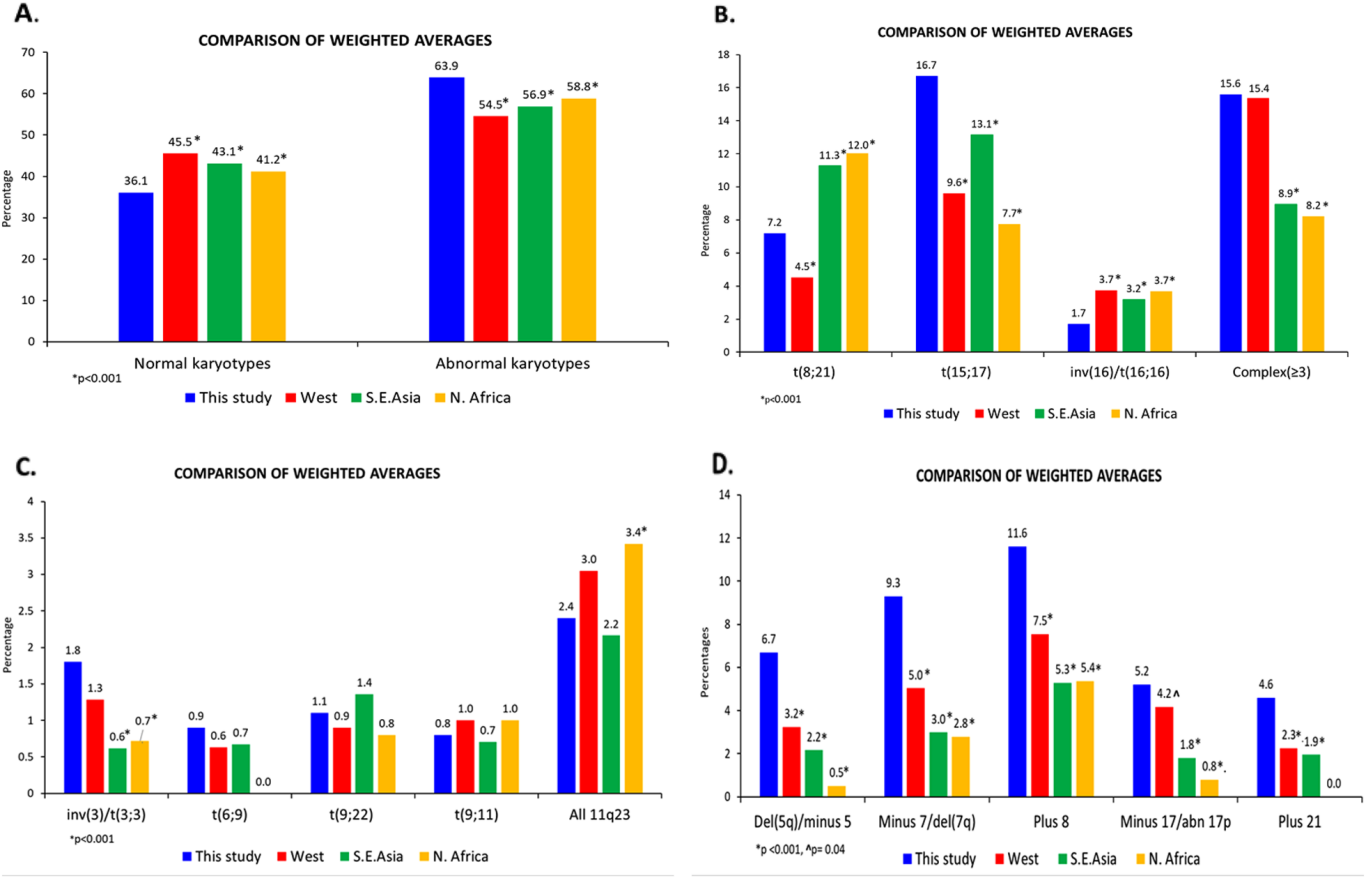
Comparison of weighted averages: **A**. Normal and abnormal karyotypes. **B**. Common RGA and complex karyotypes **C**. Other RGA. **D**. Common MRC and other abnormalities

The frequency of abnormal karyotypes in our study (64%) was comparable to the literature (55–65%) except for one report each from Malaysia (31%), Pakistan (39%) and Germany (42%); these lower frequencies could be because some abnormalities were cryptic, not detected, or excluded from the analysis [1, 12, 24, 29]. Karyotypes with ≥ 3 abnormalities were seen in 15.6%, comparable to the West but more common (*p* < 0.001) than in S.E Asia (9%) and N. Africa (8%) [9–16, 18–24, 26–28]. The frequency of complex karyotypes as defined by the WHO/ICC was reported only by Meng et al. and was lower (7.2%) than in our study (11.2%) [24]. These findings are summarised in Tables 5, 6, 7 The frequency of monosomal karyotypes determined by conventional cytogenetic analysis was comparable to the literature (8.8% vs 6–14%) [10, 15, 23, 27, 36, 37].

A comparison of the frequencies of our common abnormalities with the literature is shown in Additional files 6–8 and Fig. 4. The t (15;17), our most common abnormality (16.7%), had a higher frequency (*p* < 0.001) than in the West (9.6%) and N. Africa (7.7%) and S.E. Asia (13.1%) [10, 11, 13, 14, 16–18, 20–28]. Its frequency was low (< 4%) in Morocco and Malaysia, where it was not the most common abnormality [24, 27]. The higher frequency of the t(15;17) in most of S.E Asia (11–17% in all but one study) compared to the West could be related to the younger age at presentation, as well as genetic and ethnic factors that predispose individuals to breakage of the PML gene [22, 38]. The high frequency in our study could also be because ours is a referral centre for acute promyelocytic leukemia having initiated arsenic trioxide treatment very early in India. The t(15;17) can be overlooked in karyotypes with suboptimal morphology. FISH analysis was done in most of our patients with/suspected to have acute promyelocytic leukemia, for confirmation, and to establish baseline values for assessment of cytogenetic response post treatment.

The frequency of the t(8;21), our second most common translocation (7.2%) was higher than in the West (4.5%, *p* < 0.001) but lower than in S.E. Asia and N. Africa (11.3% and 12% respectively, *p* < 0.001) [9–28]. Its frequency was far higher in Japan (13% and 18%) and Morocco (12.5%), where it was the most common abnormality, Tunisia (12%) and a report from China (15%) [17, 19, 21, 26, 27]. We had fewer patients (1.7%, *p* < 0.001) with the inv (16)/t(16;16) than in all three regions (S.E. Asia, 3.2%, the West and N. Africa, 3.7% each) [9, 10, 13–25]. It was more than twice as common in the U.S.A, Korea, Egypt and one report each from the U.K, Australia, Japan and Malaysia (4–8%) [9, 10, 17, 19, 23, 25, 28]. The inv(16) can also be overlooked if the morphology is suboptimal, especially if cytogenetic analysis is not correlated with bone marrow morphology. FISH analysis was performed for confirmation if the bone marrow morphology showed myelomonocytic/monocytic differentiation or eosinophilia or if chromosome morphology was suggestive of this abnormality, and was negative in 14 such patients. It is possible that the frequency of our inversion (16) could be higher if FISH analysis were done in every patient.

The t(8;21) was the most common abnormality in all three other reports from South Asia (8.3–20.8%) [29–31] The t(8;21) and the inv(16) were twice as common (14.7% and 4% respectively, *p* < 0.001) as in our patients in the large study from India (1906 patients) [30].These differences could be due to the reasons mentioned above. The frequencies of t(8;21) and the inv(16)/t(16;16) were similar (21% each) in the other Indian study in which AML M2, M4 and M5 subtypes accounted for 43%,23% and 8% respectively; these unusually high frequencies which differ from all other reports could be because of referral bias, the short duration (two years) and the relatively small number (173) of patients from a single institution [31].

The inv(3/t(3;3) was more common (1.8%) than in S.E. Asia (< 1%, *p* < 0.001) and N. Africa but was comparable to the West (1.3%) [9, 10, 12, 14–16, 18–23, 27, 28]. The (9;11), the t(9;22) and the t(6;9) were comparable to the literature, although the (6;9) was not reported from N. Africa [9–11, 13, 15, 16, 18–23, 27, 28]. However, the frequency of 11q23 abnormalities (KMT2A/MLL translocations) was lower than in N. Africa (*p* = 0.02) but comparable to the West and S.E.Asia [9–28]. We had fewer NUP98 translocations than in the study from Hong Kong which was the only one that reported these abnormalities separately (0.4 vs 1.1%) [22].

Trisomy 8, monosomy 7/del 7q and del 5q/monosomy 5 were more common (*p* < 0.001) among our patients than in the other regions; their frequencies, which varied widely in the West (4–11%, 1.8–13% and 0.5–13% respectively), were lower in N. Africa and S.E.Asia (3–7%, 0.3–5% and 0.3–3% respectively) except for Singapore (monosomy 7/del 7q and del 5q/monosomy 5 in 7% each) [9–28]. The combined frequency of these three abnormalities among our patients (27.6%) was twice and almost four times as high (*p* < 0.001) as in Australia (14%) and Japan (7.5%), as reported by Nakase et al. [17]. Similarly, chromosome 5 and chromosome 7 abnormalities (16%) were three and four times more common (*p* < 0.001) than in Malaysia (5.3%) and Hong Kong (4%) [22, 25]. True monosomy 5 is reported to be uncommon in AML because evaluation with FISH/multicolour FISH or spectral karyotyping showed that the majority of such karyotypes had complex rearrangements involving chromosome 5q, with preservation of 5p [1, 39–44]. Therefore, it is possible that the frequency of our monosomy 5 could change significantly if these karyotypes were evaluated further with these techniques. However, the change in the frequency of our MK would be negligible because of the presence of one or more additional monosomies.

We had more patients with monosomy 17/del 17p than in China (*p* < 0.001) and Egypt (*p* = 0.03) [21, 28]. Trisomy 21 was more common (*p* < 0.001) than in the West and S.E.Asia; it was not reported from N. Africa [9–12, 18, 21, 22]. The higher frequency of high-risk abnormalities in our study as compared to S.E Asia could be due to the interplay of environmental factors and ethnic differences because abnormalities such as the t(15;17) have frequencies more similar to our findings than the West.

To summarise, our data confirms the lower (one to two decades) median age of patients (~ 42 years) with AML in Asia and Africa compared to Western countries. While the frequency of our abnormal karyotypes is comparable to the literature, there are similarities and differences with respect to the common abnormalities. We had more patients with the t(8;21) than in the West, but fewer than in the rest of Asia and Africa. Other major differences included higher frequencies of the t(15;17), trisomy 8 and trisomy 21, and a lower frequency of the inv(16). The high-risk abnormalities such as monosomy 7 and del 5q/monosomy 5 were also more common than in other regions while the inv(3)/t(3;3) and monosomy 17/del 17p had higher frequencies than in S.E. Asia and N. Africa; these abnormalities were more common in younger patients (≤ 60 years) compared to the West. A limitation of this report is the lack of molecular profile of these patients who were evaluated over a long period of time when such assessment was not always feasible. These differences in the median age and frequency of AML-associated cytogenetic abnormalities in different parts of the world could reflect ethnic/genetic differences in the susceptibility to environmental agents associated with leukemogenesis and the response to genetic damage. More detailed epidemiological studies of possible environmental exposure coupled with next-generation sequencing and emerging technologies such as optical genome mapping to look for germline abnormalities that could predispose to these conditions would help to better understand why some chromosomal abnormalities are more common than others in different geographic regions and ethnic groups.

### Supplementary Information

**Additional file 1**. Comparison of age in normal and abnormal karyotypes.

**Additional file 2**. Other 11q23 (KMT2A / MLL), NUP98 and raretranslocations in AML.

**Additional file 3**. Other (non-RGA) translocations.

**Additional file 4**. Abnormalities frequently seen in complex karyotypes.

**Additional file 5**. Distribution of abnormalities according to cytogenetic risk groups.

**Additional file 6**. Raw data and statistical analysis for comparison with reports from the West.

**Additional file 7**. Raw data and statistical analysis for comparison of our findings with reports from S.E Asia.

**Additional file 8**. Raw data and statistical analysis for comparison with reports from N.Africa.

## Data Availability

The datasets used and/or analysed during the current study are available from the corresponding author on reasonable request.

## Abbreviations

abn: Abnormality/abnormalities
ACA: Additional cytogenetic abnormalities
AML: Acute myeloid leukemia
AML-RGA: AML with recurrent genetic abnormalities
AML-MRC: AML with myelodysplasia-related cytogenetic changes
CK: Complex karyotype(s)
del: Deletion
CBFB: Core-binding factor subunit beta
ELN: European LeukemiaNet
ISH: Fluorescence in situ hybridization
ICC: International Consensus Classification of Acute Myeloid Leukemia
ISCN: International System for Human Cytogenomic Nomenclature
i: Isochromosome
ider: Isoderivative
inv: Inversion
M:F ratio: Male:female ratio
MK: Monosomal karyotypes
MRC: Myelodysplasia-related cytogenetic abnormalities
N.Africa: North Africa
PML: Promyelocytic leukemia nuclear body scaffold
RARA: Retinoic acid receptor alpha
RGA: Recurrent genetic abnormalities
S.E.Asia: South-East Asia
t: Translocation
UK: United Kingdom
USA: United States of America
WHO: World Health Organisation
