# Supplementary material for: Cytogenetic profile of 1791 adult acute myeloid leukemia in India
Source: Mol Cytogenet. 2023 Sep 16;16:24. doi: 10.1186/s13039-023-00653-1 (PMC10504794; doi:10.1186/s13039-023-00653-1)
Supplement: Supplementary file 1 — Additional file 1. Comparison of age in normal and abnormal karyotypes. [file 13039_2023_653_MOESM1_ESM.docx]

**Additional File 1. Supplementary Table 1. Comparison of age in normal and abnormal karyotypes. SPSS output : Anova**

| **ANOVA** | | | | | | | |  |  |
| --- | --- | --- | --- | --- | --- | --- | --- | --- | --- |
| age | | | | | | | |  |  |
|  | Sum of Squares | | df | Mean Square | F | Sig. | |  |  |
| Between Groups | 15232.695 | | 4 | 3808.174 | 19.006 | .000 | |  |  |
| Within Groups | 357855.447 | | 1786 | 200.367 |  |  | |  |  |
| Total | 373088.142 | | 1790 |  |  |  | |  |  |
| **Multiple Comparisons** | | | | | | | | | |
| Dependent Variable: age | | | | | | | | | |
| Bonferroni | | | | | | | | | |
| (I) NoofabnNkt1single2double3threeormore4n279 | | (J) NoofabnNkt1single  2double3threeormore4n279 | | Mean Difference  (I-J) | Std. Error | Sig. | 95% Confidence Interval | | |
|  |  |  |  |  |  |  | Lower Bound | | Upper Bound |
| Normal karyotype | | Single abnormality | | 4.424^*^ | .815 | .000 | 2.14 | | 6.71 |
|  |  | Two abnormalities | | 5.384^*^ | .990 | .000 | 2.60 | | 8.17 |
|  |  | Three or more abnormalities | | 9.283^*^ | 1.687 | .000 | 4.54 | | 14.02 |
|  |  | Complex karyotype | | -1.360 | 1.145 | 1.000 | -4.58 | | 1.86 |
| Single abnormality | | Normal karyotype | | -4.424^*^ | .815 | .000 | -6.71 | | -2.14 |
|  |  | Two abnormalities | | .959 | 1.012 | 1.000 | -1.88 | | 3.80 |
|  |  | Three or more abnormalities | | 4.858^*^ | 1.700 | .043 | .08 | | 9.64 |
|  |  | Complex karyotype | | -5.785^*^ | 1.164 | .000 | -9.06 | | -2.51 |
| Two abnormalites | | Normal karyotype | | -5.384^*^ | .990 | .000 | -8.17 | | -2.60 |
|  |  | Single abnormality | | -.959 | 1.012 | 1.000 | -3.80 | | 1.88 |
|  |  | Three or more abnormalities | | 3.899 | 1.791 | .296 | -1.13 | | 8.93 no diff bc number less than single, and similar tp three |
|  |  | Complex karyotype | | -6.744^*^ | 1.293 | .000 | -10.38 | | -3.11 |
|  | |  | |  |  |  |  | |  |
|  | |  | | **Multiple Comparisons contd…..** | | | | | |
| Three or more abnormalities | | Normal karyotype | | -9.283^*^ | 1.687 | .000 | -14.02 | | -4.54 |
|  |  | Single abnormality | | -4.858^*^ | 1.700 | .043 | -9.64 | | -.08 |
|  |  | Two abnormalites | | -3.899 | 1.791 | .296 | -8.93 | | 1.13 no diff bc number less than single, and similar tp three |
|  |  | Complex karyotype | | -10.643^*^ | 1.881 | .000 | -15.93 | | -5.36 |
| Complex karyotype | | Normal karyotype | | 1.360 | 1.145 | 1.000 | -1.86 | | 4.58 |
|  |  | Single abnormality | | 5.785^*^ | 1.164 | .000 | 2.51 | | 9.06 |
|  |  | Two abnormalites | | 6.744^*^ | 1.293 | .000 | 3.11 | | 10.38 |
|  |  | Three or more abnormalities | | 10.643^*^ | 1.881 | .000 | 5.36 | | 15.93 |
| *. The mean difference is significant at the 0.05 level. | | | | | | | | | |

| **ANOVA** | | | | | | |  |  |
| --- | --- | --- | --- | --- | --- | --- | --- | --- |
| age | | | | | | |  |  |
|  | Sum of Squares | | df | Mean Square | F | Sig. |  |  |
| Between Groups | 8426.919 | | 2 | 4213.459 | 20.753 | <.001 |  |  |
| Within Groups | 362821.314 | | 1787 | 203.034 |  |  |  |  |
| Total | 371248.232 | | 1789 |  |  |  |  |  |
| **Multiple Comparisons** | | | | | | | | |
| Dependent Variable: age  Bonferroni | | | | | | | | |
| (I) Abnormality_categ | | (J) Abnormality_categ | | Mean Difference (I-J) | Std. Error | Sig. | 95% Confidence Interval | |
|  |  |  |  |  |  |  | Lower Bound | Upper Bound |
| Normal | | 1 to 2 abnormality | | 4.693^*^ | .741 | <.001 | 2.92 | 6.47 |
|  |  | >=3 abnormality | | 1.591 | 1.021 | .358 | -.86 | 4.04 |
| 1 to 2 abnormality | | Normal | | -4.693^*^ | .741 | <.001 | -6.47 | -2.92 |
|  |  | >=3 abnormality | | -3.102^*^ | .981 | .005 | -5.45 | -.75 |
| >=3 abnormality | | Normal | | -1.591 | 1.021 | .358 | -4.04 | .86 |
|  |  | 1 to 2 abnormality | | 3.102^*^ | .981 | .005 | .75 | 5.45 |
| *. The mean difference is significant at the 0.05 level. | | | | | | | | |
