# Supplementary material for: Cytogenetic profile of 1791 adult acute myeloid leukemia in India
Source: Mol Cytogenet. 2023 Sep 16;16:24. doi: 10.1186/s13039-023-00653-1 (PMC10504794; doi:10.1186/s13039-023-00653-1)
Supplement: Supplementary file 2 — Additional file 2. Other 11q23 (KMT2A / MLL), NUP98 and raretranslocations in AML. [file 13039_2023_653_MOESM2_ESM.docx]

| **Additional File 2. Supplementary Table 2.** | | | | | | | | | | | | | |
| --- | --- | --- | --- | --- | --- | --- | --- | --- | --- | --- | --- | --- | --- |
| **Supplementary Table 2: Other 11q23 (KMT2A / MLL), NUP98 and rare translocations in AML** | | | | | | | | | | | | | |
|  | **No. of patients, n(%)** | | | **Age** | **Age distribution, years, n (%)** | | | | | | **Distribution in KTs, n (%)** | | |
| **Abnormality** | **Total** | **M** | **F** | **Median(range)** | **18-20y** | **21-29y** | **30-39** | **40-49y** | **50-59** | **60-69*** | **Single** | **Double** | **≥3** |
| **Other 11q23 (KMT2A / MLL) translocations, n=28** | | | | | | | | | | | | | |
| t(6;11)(q27;q23) | 9(0.5) | 5(55.6) | 4(44.4) | 34(23-65) | 0(0) | **3(33.3)** | **2(22.2)** | **2(22.2)** | 0(0) | **2(22.2)** | 3(33.3) | 3(33.3) | 3(33.3) |
| t(10;11)(p11.2~p12;q23) | 5(0.3) | 4(80) | 1(20) | 28(22-47) | 0(0) | **3(60)** | 0(0) | 2(40) | 0(0) | 0(0) | 4(80) | 1(20) | 0(0) |
| t(10;11)(q21;q23) | 1(0.06) | 1(100) | 0(0) | 39 | 0(0) | 0(0) | **1(100)** | 0(0) | 0(0) | 0(0) | 1(100) | 0(0) | 0(0) |
| t(11;19)(q23;p13.1) | 4(0.2) | 3(75) | 1(25) | 39(19-59) | **1(25)** | **1(25)** | 0(0) | 0(0) | **2(50)** | 0(0) | 4(100) | 0(0) | 0(0) |
| t(11;19)(q23;p13.3) | 3(0.2) | 1(33.3) | 2(66.7) | 37(32-65) | 0(0) | 0(0) | **2(66.7)** | 0(0) | 0(0) | **1(33.3)** | 2(66.7) | 1(33.3) | 0(0) |
| t(11;17)(q23;q25) | 3(0.2) | 1(33.3) | 2(66.7) | 50(47-55) | 0(0) | 0(0) | 0(0) | **1(33.3)** | **2(66.6)** | 0(0) | 3(100) | 0(0) | 0(0) |
| t(X;11)(q24;q23) | 2(0.1) | 2(100) | 0(0) | 35(18,51) | **1(50)** | 0(0) | 0(0) | 0(0) | **1(50)** | 0(0) | 0(0) | 3(100) | 0(0) |
| t(1;11)(q21;q23) | 1(0.06) | 0(0) | 1(100) | 50 | 0(0) | 0(0) | 0(0) | 0(0) | **1(100)** | 0(0) | 0(0) | 1(100) | 0(0) |
| **NUP98 translocations, n=8** | | | | | | | | | | | | | |
| t(7;11)(p15;p15) | 4(0.2) | 2(50) | 2(50) | 41(22-67) | 0(0) | **1(25)** | **1(25)** | **1(25)** | 0(0) | **1(25)** | 2(50) | 1(25) | 1(25) |
| t(11;20)(p15;q12) | 2(0.1) | 0(0) | 2(100) | 47.5(41,54) | 0(0) | 0(0) | 0(0) | 1(50) | 1(50) | 0(0) | 2(100) | 0(0) | 0(0) |
| t(2;11)(q31;p15) | 1(0.06) | 1(100) | 0(0) | 49 | 0(0) | 0(0) | 0(0) | 1(100) | 0(0) | 0(0) | 0(0) | 0(0) | 1(100) |
| t(11;12)(p15;q13) | 1(0.06) | 0(0) | 1(100) | 35 | 0(0) | 0(0) | 1(100) | 0(0) | 0(0) | 0(0) | 0(0) | 0(0) | 1(100) |
| **Other rare translocations, n=10** | | | | | | | | | | | | | |
| t(10;11)(p12;q14) | 6(0.3) | 2(33.3) | 4(66.7) | 26(20-33) | **1(16.7)** | **3(50)** | **2(33.3)** | 0(0) | 0(0) | 0(0) | 3(50) | 1(16.7) | 2(33.3) |
| t(8;16)(p11;p13) | 1(0.06) | 0(0) | 1(100) | 31 | 0(0) | 0(0) | **1(100)** | 0(0) | 0(0) | 0(0) | 1(100) | 0(0) | 0(0) |
| t(16;21)(p11.2;q22) | 1(0.06) | 1(100) | 0(0) | 31 | 0(0) | 0(0) | **1(100)** | 0(0) | 0(0) | 0(0) | 0(0) | 1(100) | 0(0) |
| t(1;3)(p36.3;q21.2) | 1(0.06) | 1(100) | 0(0) | 54 | 0(0) | 0(0) | **1(100)** | 1(100) | 0(0) | 0(0) | 0(0) | 0(0) | 1(100) |
| t (3;5)(q25;q35) | 1(0.06) | 0(0) | 1(100) | 35 | 0(0) | 0(0) | **1(100)** | 0(0) | 0(0) | 0(0) | 0(0) | 1(100) | 0(0) |
| *none ≥70 |  |  |  |  |  |  |  |  |  |  |  |  |  |
