# Supplementary material for: Cytogenetic profile of 1791 adult acute myeloid leukemia in India
Source: Mol Cytogenet. 2023 Sep 16;16:24. doi: 10.1186/s13039-023-00653-1 (PMC10504794; doi:10.1186/s13039-023-00653-1)
Supplement: Supplementary file 3 — Additional file 3. Other (non-RGA) translocations. [file 13039_2023_653_MOESM3_ESM.docx]

| **Additional File 3: Supplementary Table 3: Other (non-RGA) translocations** | | | |
| --- | --- | --- | --- |
| No. | Age | Sex | Karyotype |
| 1 | 49 | M | 46~48,XY,+Y,t(1;16)(p31;q24),+22[cp19] |
| 2 | 46 | F | 48,XX,t(1;21)(p36;q22),+8,+21[19]/45,XX,-21[1 cell] |
| 3 | 52 | M | 43~45,XY,t(4;12)(q12;p13),-5,del(5)(q13q33),add(7)(q36),del(13)(q14),-17,del(17)(p12),18,+19,20[cp16]/ 46,XY[4] |
| 4 | 33 | M | 46,XY,t(5;12)(q33,p13.2)[4]/45,idem,-20[5]/46,XY[11] |
| 5 | 60 | F | 45,XX,t(1;3)(q43;p22),t(3;5)(q21;q15),-7[20] |
| 6 | 72 | M | 46,XY,t(3;9)(q21 or q26;q33 or 34)[11]/47,idem,del(18)(q22)[9] |
| 7 | 34 | M | 46,XY,t(3;12)(q21;q24),der(16)t(16;17)(q24;q2?2?3),-17,+mar[17]/46,XY[3] |
| 8 | 21 | M | 48,XY,t(3;12)(q12;p13),+8,+19[20] |
| 9 | 48 | M | 45~47,XY,del(5)(q13q33),t(6;12)(q14;p13),+8,der(14)t(11;14)(q13;q32),der(16)?r(16)del(20)(q12)[cp20] |
| 10 | 72 | M | 46, XY, del(5)(q31q34), del(8)(?q13?q22), t(8;12)(q22;p13)[cp 12] |
| 11 | 30 | F | 46,XX,t(2;5)(p12;q35)[20] |
| 12 | 35 | F | 46,XX,t(5;14)(q35;q11) [4] / 46,idem,-21,+22 [2] /47,idem,+22 [12] / 46,XX [2] |
| 13 | 40 | M | 47,XY,dup(3)(q21q24),t(5;15)(q13;p11),+8[15]/46,XY[5] |
| 14 | 60 | F | 46,X,der(X)?del(X)(q23),t(5;11)(q13;q21 or q23)[cp15]/46,XX[5] |
| 15 | 55 | M | 45,XY, t(1;7)(q10;p10), -7 [12] / 46, idem, +21 [3] / 49, idem, +der 7, t(1;7)(q10;p10), +8, +13, +21 [3] |
| 16 | 30 | F | 45,X,-X,t(7;10;8)(q11.2 or q21;p13 or p15;q22)[3]/46,idem,1dmin or +mar[15]/46,XX[2] |
| 17 | 33 | M | 46,XY,t(7;17)(q21;p13)[19]/46,XY[1 cell] |
| 18 | 60 | M | 47,XY,t(7;19)(q21;p13.3 or q13.3),+8[18]/46,XY[2] |
| 19 | 72 | M | 45,XY,-7[6]/45,idem,t(2;21)(p12;q22)[8]/46,XY[6] |
| 20 | 20 | F | 46,XX,t(1;2)(p36;p21),add(19)(p13.3)[19]/46,XX[1cell] |
| 21 | 21 | F | 45,X,-X,t(1;2)(p34 or p36.1;p21 or p23)[5]/46,XY[15] |
| 22 | 28 | F | 46,XX,t(1;3)(p21;p25),t(2;3)(p16;p21),del(5)(q13q23),add(6)(p25),-9,t(11;18)(q2?3?4;p11.2),del(13)(q21q22),-16,-17,-19[17]/88~92,idem,-5,+6,-13[3] |
| 23 | 45 | M | 46,XY,t(1;11)(p36;q13~14) [10] / 46,XY [10] |
| 24 | 47 | M | 44~45,XY,del(1)(p21),t(1;11;19)(p11;q25;q21),der(5)?t(?;5)(?;p14),-10[cp17]/46,XY[2] |
| 25 | 33 | M | 46,XY,t(2;10)(q?31q34;q22)[20] |
| 26 | 50 | M | 46,XY,t(2;11)(q37;q21),del(12)(p13)[20] |
| 27 | 49 | M | 46,XY,t(3;13)(p25 or q27;q14)[20] |
| 28 | 72 | M | 41~44,XY,t(3;17)(p21;q11),-5,del(7)(q21q31),add(12)(p13)?der(12)t(12;19)(p13;?p13?q13),  der(15),-16,-17,-19,+1~2mar[cp19] |
| 29 | 42 | F | 46,XX,t(4;9)(q33;q22)[20] |
| 30 | 60 | M | 46,XY,t(5;16)(p10;q10)[20] |
| 31 | 65 | M | 46,XY,t(6;12)(q25;q21)[17]/46,XY[3] |
| 32 | 44 | M | 46,XY,t(9;17)(q31q33;q21)[17]/46,XY[2] |
| 33 | 29 | F | 71~80,XXX,+3,+6,+8,+9,t(10;12)(p10;q10),+t(10;12)(p10;q10),+11,+14,+19,+20,+21[cp20] |
| 34 | 20 | F | 46,XX,t(10;13;22)(p11.2;q14;q13)[19]/46,XX[1cell] |
| 35 | 63 | F | 46,XX,del(5)(q31q34),t(11;12)(q12;q24.1),del(12)(q13),add(22)(q13) or der(22)t(?5;22)(?q32;q13)[cp20] |
| 36 | 37 | F | 47,XX,-6,+8,t(11;14)(p11.2;q11.2 or q13 or 22),del(12)(p13),add(15)(p11.2),+mar[11]/46,XX[9] |
| 37 | 24 | M | 46,XY,t(12;12)(q14;q24.3)[4]/46,XY[22] |
| 38 | 20 | M | 46,XY,t(13;17)(q11;q25)[19] |
| 39 | 52 | F | 46,XX,t(14;17)(q11.2;q25)[11]/46,idem,t(X;18)(p22;q21)[5]/47,idem,+8[4] |
| 40 | 45 | F | 46,X,t(X;10)(q11,q21),del(1)(p?35 ?36),t(10;11)(q13-15;q21) [20] |
