# Supplementary material for: Cytogenetic profile of 1791 adult acute myeloid leukemia in India
Source: Mol Cytogenet. 2023 Sep 16;16:24. doi: 10.1186/s13039-023-00653-1 (PMC10504794; doi:10.1186/s13039-023-00653-1)
Supplement: Supplementary file 4 — Additional file 4. Abnormalities frequently seen in complex karyotypes. [file 13039_2023_653_MOESM4_ESM.docx]

|  | | | | | | | |
| --- | --- | --- | --- | --- | --- | --- | --- |
| **Additional File 4: Supplementary Table 4. Complex karyotypes** | | | | | | | |
|  | **Minus 7** | **del 7q** | | **del 5q** | **Minus 5** | **Minus 17** | **Minus 18** |
| **All karyotypes** | **49** | **18** | | **40** | **53** | **51** | **42** |
| Minus 7 |  | 5 | | 15 | 25 | 15 | 17 |
| del 7q | 5 |  | | 5 | 8 | 7 | 4 |
| del 5q | 15 | 5 | |  | 9 | 13 | 9 |
| Minus 5 | 25 | 8 | | 9 |  | 25 | 21 |
| Minus 17 | 15 | 7 | | 13 | 25 |  | 24 |
| Minus 18 | 17 | 4 | | 9 | 21 | 24 |  |
|  | | | | | | | |
| Minus 5, del 5q | 3 | 3 | |  |  | 5 | 5 |
| Minus 5, del 7q | 2 |  | | 3 |  | 2 | 1 |
| Minus 5, minus 17 | 8 | 4 | | 5 |  |  | 15 |
| Minus 5, minus 18 | 9 | 1 | | 3 |  | 15 |  |
| Minus 5, minus 17, minus 18, n=15 | 5 | 1 | | 2 |  |  |  |
| del 5q, del 7q | 2 |  | |  | 3 | 2 | 1 |
| del 5q, minus 18 | 2 | 1 | |  | 3 | 5 |  |
| del 5q, minus 17 | 3 | 2 | |  | 5 |  | 9 |
| del 5q, minus 17, minus 18, n=5 | 2 | 0 | |  | 2 |  |  |
| del 5q, del 7q, minus 18, n=1 | 0 |  | |  | 0 | 0 |  |
| del 5q, minus 5, minus 17, minus 18, n=2 | 1 | 0 | |  |  |  |  |
| del 7q, minus 5, minus 17, minus 18, n=1 | 0 |  | | 0 |  |  |  |
| del 7q, minus 17 | 2 |  | | 2 | 4 |  | 2 |
| del 7q, minus 18 | 2 |  | | 1 | 1 | 2 |  |
| Minus 17, minus 18 | 10 | 2 | | 5 | 15 |  |  |
|  | | | | | | |  |
| **Abnormalities of chromosomes 5 and/or 7 in CK** | | | **Present** | | **Absent** | **Total** |  |
| No. of CK | | | 109 | | 91 | 200 |  |
| Median age (range), years | | | 53 (18-82) | | 40 (18-72) |  |  |
| No. of abnormalities, median (range) | | | 10 (3-28) | | 4 (3-23) |  |  |
| *Breakdown of chromosome 5 and 7 abnormalities in CK* | | | | | | |  |
| del 5q | | |  | |  | 40 |  |
| del 7q | | |  | |  | 18 |  |
| Minus 5 | | |  | |  | 53 |  |
| del 5q without minus 5 | | | 31 | |  |  |  |
| Minus 7 | | |  | |  | 49 |  |
| del 7q without minus 7 | | | 13 | |  |  |  |
| Minus 5 without minus 7 | | | 30 | |  |  |  |
| Minus 7 without minus 5 | | | 26 | |  |  |  |
| Minus 5 and minus 7 | | | 23 | |  |  |  |
|  | | |  | |  |  |  |
| Minus 5 without minus 7, del 7q, del 5q | | | 19 | |  |  |  |
| Minus 7 without minus 5, del 7q, del 5q | | | 17 | |  |  |  |
| del 5q without minus 5, minus 7, del 7q | | | 23 | |  |  |  |
| del 7q without minus 5, minus 7, del 5q | | | 5 | |  |  |  |
|  | | |  | |  |  |  |
| **Additional File 4: Supplementary Table 4. Complex karyotypes contd….** | | | | | | |  |
| **Other abnormalities** | | | **Present** | | **Absent** | **Total** |  |
| Minus 17 | | | 40 | | 11 | 51 |  |
| Minus 18 | | | 34 | | 8 | 42 |  |
| Trisomy 8 | | | 31 | | 30 | 61 |  |
| Trisomy 21 | | | 20 | | 23 | 43 |  |
