# Supplementary material for: Cytogenetic profile of 1791 adult acute myeloid leukemia in India
Source: Mol Cytogenet. 2023 Sep 16;16:24. doi: 10.1186/s13039-023-00653-1 (PMC10504794; doi:10.1186/s13039-023-00653-1)
Supplement: Supplementary file 5 — Additional file 5. Distribution of abnormalities according to cytogenetic risk groups. [file 13039_2023_653_MOESM5_ESM.docx]

| **Additional File5. Supplementary Table 5. Distribution of karyotypes according to cytogenetic risk groups.** | | | | |
| --- | --- | --- | --- | --- |
| **Cytogenetic risk group** | **Overall, n (%)** |  |  | **Total, n (%)**** |
| ***Favorable risk*** |  |  |  | **459 (25.6)** |
| t(15;17)(q22;q21) | 299(16.7) |  |  |  |
| t(8;21)(q22;q22.1 | 129(7.2) |  |  |  |
| inv(16)(p13.1q22) or t(16;16)(p13.1;q22) | 31(1.7) |  |  |  |
|  |  |  |  |  |
| ***Intermediate risk*** |  |  |  | **958 (53.5)** |
| *Normal karyotypes* | 646 (36.1) |  |  | 646 (36.1) |
| *Abnormal karyotypes* |  |  |  | **312(17.4)** |
| *RGA* |  |  |  | *31(1.7)* |
| t(9;11)(p21.3;q23.3) | 14(0.8) |  |  |  |
| t NUP98 | 8(0.4) |  |  |  |
| Rare t | 9(0.5) |  |  |  |
| *MRC* |  |  |  | *121(6.8)* |
|  |  | Solitary | Double* |  |
| Plus 8 | 208 (11.6) | 67 | 20 | 87(4.9) |
| del 7q | 45(2.5) | 11 | 7 | 18 (1.0) |
| del 11q | 14(0.8) | 0 | 1 | 1(0.06) |
| del /add/t 12p | 34(1.9) | 3 | 7 | 10(0.6) |
| Minus 13 /del 13q | 35(2) | 0 | 1 | 1(0.06) |
| del 20q | 11(0.6) | 1 | 3 | 4(0.2) |
| Other^ |  |  |  | 160(15.7) |
| del 9q | 60(3.4) | 14 | 3 | 17(0.9) |
| Plus 21 | 83(4.6) | 16 | 4 | 20(1.1) |
| Plus 4 | 54(3) | 10 | 9 | 19(1.1) |
| Other less frequent abnormalities |  |  |  | 160 (8.9) |
|  |  |  |  |  |
| **Adverse risk** |  |  |  | **374(21)** |
| *RGA* |  |  |  | *96(5.4)* |
| inv(3)(q21.3q26.2) or t(3;3)(q21.3;q26.2) | 32(1.8) |  |  |  |
| t(6;9)(p23.3;q34.1)/DEK::NUP214 | 16(0.9) |  |  |  |
| t(9;22)(q34.1;q11.2)/BCR::ABL1 | 19(1.1) |  |  |  |
| t(v;11q23.3)/KMT2A-rearranged# | 28(1.6) |  |  |  |
| t(8;16)(p11.2;p13.3)/KAT6A::CREBBP | 1(0.06) |  |  |  |
| *MRC* |  |  |  | *78(4.4)* |
|  |  | Solitary | Double* |  |
| del(5q) | 65(3.6) | 10 | 6 | 16(0.9) |
| Minus 5 | 122(6.8) | 0 | 0 | 0 |
| Minus 7 | 122(6.8) | 30 | 18 | 48(2.7) |
| Minus 17/abn(17p) | 94(5.2) | 7 | 6 | 14(0.8) |
| Complex karyotypes (CK), WHO/ICC^ | 200(11.2) |  |  | 200(11.2) |
| Monosomal karyotypes | 158(8.8) |  |  |  |
| *Double, without RGA or other adverse; **excluding those associated with CK; ^excluding RGA ≥3; ^ only the most frequent abnormalities (frequency ≥3%) are shown. | | | | |
