# Supplementary material for: Cytogenetic profile of 1791 adult acute myeloid leukemia in India
Source: Mol Cytogenet. 2023 Sep 16;16:24. doi: 10.1186/s13039-023-00653-1 (PMC10504794; doi:10.1186/s13039-023-00653-1)
Supplement: Supplementary file 6 — Additional file 6. Raw data and statistical analysis for comparison with reports from the West. [file 13039_2023_653_MOESM6_ESM.docx]

| **Additional file 6: Supplementary Table 6: Raw data and statistical analysis for comparison of our findings with reports from the West** | | | | | | | | | | | | | |
| --- | --- | --- | --- | --- | --- | --- | --- | --- | --- | --- | --- | --- | --- |
| **Country** | **This study**  **% (95% CI)** | **Germany** | **USA** | **UK** | | **Spain** | **Sweden** | **Germany** | | **Australia** | | **Total size &**  **weighted**  **proportions** |  |
|  |  | **Bacher** | **Byrd** | **Sanderson** | **Grimwade** | **Spain** | **Lazarevic** | **Creutzig** | | **Nakase** | **Gangatharan** |  |  |
|  |  |  | **Normal karyotypes** | | |  |  |  | |  |  |  | **P value** |
| KT analysed | 1791 | 2235 | 1213 | 1192 | 5876 | 1129 | 1893 | 4372 | | 230 | 710 | 18850 |  |
| Normal KT, % | 36.1 | 58 | 48 | 45 | 41 | 37 | 43 | 49 | | 44 | 39 |  |  |
| Weight |  | 0.1 | 0.06 | 0.06 | 0.3 | 0.06 | 0.1 | 0.2 | | 0.01 | 0.04 |  |  |
| Proportion | 36.1 (33.9, 38.3) | 6.9 | 3.1 | 2.8 | 12.8 | 2.2 | 4.3 | 11.4 | | 0.5 | 1.5 | 45.5 | <0.001 |
|  |  |  |  |  |  |  |  |  | |  |  |  |  |
|  |  |  | **Abnormal karyotypes** | | |  |  |  | |  |  |  |  |
| KT analysed | 1791 | 2235 | 1213 | 1192 | 5876 | 1129 | 1893 | 4372 | | 230 | 710 | 18850 |  |
| Abnormal KT, % | 64 | 42 | 52 | 55 | 59 | 63 | 57 | 51 | | 56 | 61 |  |  |
| Weight |  | 0.1 | 0.06 | 0.06 | 0.3 | 0.06 | 0.1 | 0.2 | | 0.01 | 0.04 |  |  |
| Proportion | 64(61.8, 66.2) | 5.0 | 3.3 | 3.5 | 18.4 | 3.8 | 5.7 | 11.8 | | 0.7 | 2.3 | 54.5 | <0.001 |
|  |  |  |  |  |  |  |  |  | |  |  |  |  |
|  |  |  | **inv(3) /t(3;3)** | | |  |  |  |  | |  |  |  |
| KT analysed | 1791 | 2235 | 1213 |  | 5876 | 1129 | 1893 | 4372 | |  | 710 | 17428 |  |
| inv(3) /t(3;3), % | 1.8 | 1.6 | 1 | NA | 1 | 3.2 | 1 | 1.3 | | NA | 0.7 |  |  |
| Weight |  | 0.1 | 0.07 |  | 0.3 | 0.06 | 0.1 | 0.3 | |  | 0.04 |  |  |
| Proportion | 1.8 (1.2, 2.4) | 0.2 | 0.07 |  | 0.3 | 0.2 | 0.1 | 0.3 | |  | 0.03 | 1.3 | 0.062 |
|  |  |  |  |  |  |  |  |  | |  |  |  |  |
|  |  |  | **Del(5q)/minus 5** | | |  |  |  |  | |  |  | |
| KT analysed | 1791 | 2235 | 1213 | 1192 | 5876 | 1129 | 1893 | 4372 | | NA | 710 | 18620 |  |
| Del 5q/minus 5, % | 6.7 | 1.7 | 5.6 | 7 | 4 | 9.1 | 13 | 1.5 | |  | 0.5 |  |  |
| Weight |  | 0.1 | 0.07 | 0.06 | 0.3 | 0.06 | 0.1 | 0.2 | |  | 0.04 |  |  |
| Proportion | 6.7 (5.5, 7.9) | 0.2 | 0.4 | 0.4 | 1.3 | 0.6 | 1.3 | 0.4 | |  | 0.02 | 4.5 | <0.001 |
|  |  |  |  |  |  |  |  |  | |  |  |  |  |
|  |  |  | **t(6;9)** | | |  |  |  |  | |  |  |  |
| KT analysed | 1791 |  | 1213 |  | 5876 |  | 1893 | 4372 | |  |  | 13354 |  |
| t(6;9), % | 0.9 | NA | 0.7 | NA | 1 |  | 0.2 | 0.3 | | NA | NA |  |  |
| Weight |  |  | 0.09 |  | 0.4 |  | 0.1 | 0.3 | |  |  |  |  |
| Proportion | 0.9 (0.4, 1.3) |  | 0.06 |  | 0.4 |  | 0.03 | 0.1 | |  |  | 0.6 | 0.1 |
| **Additional file 6: Supplementary Table 6: Raw data and statistical analysis for comparison with reports from the West contd….** | | | | | | | | | | | | | |
| **Country** | **This study**  **% (95% CI)** | **Germany** | **USA** | **UK** | | **Spain** | **Sweden** | **Germany** | | **Australia** | | **Total size &**  **weighted**  **proportions** |  |
|  |  | **Bacher** | **Byrd** | **Sanderson** | **Grimwade** | **Spain** | **Lazarevic** | **Creutzig** | | **Nakase** | **Gangatharan** |  |  |
|  |  |  | **Minus 7/del(7q)** | | |  |  |  | |  |  |  | **P value** |
| KT analysed | 1791 | 2235 | 1213 | 1192 | 5876 | 1129 | 1893 | 4372 | |  | 710 | 18620 |  |
| Minus 7/del 7q, % | 9.3 | 2.6 | 5.5 | 5 | 5.2 | 8.6 | 13 | 1.8 | | NA | 3.5 |  |  |
| Weight |  | 0.1 | 0.07 | 0.06 | 0.3 | 0.06 | 0.1 | 0.2 | |  | 0.04 |  |  |
| Proportion | 9.3 (8.0, 10.6) | 0.3 | 0.4 | 0.3 | 1.6 | 0.5 | 1.3 | 0.4 | |  | 0.1 | 5.0 | <0.001 |
|  |  |  |  |  |  |  |  |  | |  |  |  |  |
|  |  |  | **Plus 8** | | |  |  |  | |  |  |  |  |
| KT analysed | 1791 | 2235 | 1213 | 1192 | 5876 | 1129 | 1893 | 4372 | |  | 710 | 18620 |  |
| Plus 8, % | 11.6 | 5.7 | 10.1 | 6 | 10 | 11.4 | 4.3 | 5.6 | | NA | 5.7 |  |  |
| Weight |  | 0.1 | 0.07 | 0.06 | 0.3 | 0.06 | 0.1 | 0.2 | |  | 0.04 |  |  |
| Proportion | 11.6 (10.1, 13.1) | 0.7 | 0.7 | 0.4 | 3.2 | 0.7 | 0.4 | 1.3 | |  | 0.2 | 7.5 | <0.001 |
|  |  |  |  |  |  |  |  |  | |  |  |  |  |
|  |  |  | **t(8;21)** | | |  |  |  |  | |  |  |  |
| KT analysed | 1791 | 2235 | 1213 | 1192 | 5876 | 1129 | 1893 | 4372 | | 230 | 710 | 18850 |  |
| t(8;21), % | 7.2 | 4.3 | 6.7 | 4 | 7 | 2.7 | 1.9 | 2.6 | | 5.2 | 3.1 |  |  |
| Weight |  | 0.1 | 0.06 | 0.06 | 0.3 | 0.06 | 0.1 | 0.2 | | 0.01 | 0.04 |  |  |
| Proportion | 7.2 (6.0, 8.4) | 0.5 | 0.4 | 0.3 | 2.2 | 0.2 | 0.2 | 0.6 | | 0.06 | 0.12 | 4.5 | <0.001 |
|  |  |  |  |  |  |  |  |  | |  |  |  |  |
|  |  |  | **t(9;22)** | | |  |  |  |  | |  |  | |
| KT analysed | 1791 |  | 1213 | 1192 | 5876 |  |  |  | |  | 710 | 8981 |  |
| t(9;22), % | 1.1 | NA | 0.8 | 1 | 1 | NA | NA | NA | | NA | 0.1 |  |  |
| Weight |  |  | 0.1 | 0.1 | 0.7 |  |  |  | |  | 0.08 |  |  |
| Proportion | 1.1 (0.6, 1.6) |  | 0.1 | 0.1 | 0.7 |  |  |  | |  | 0.01 | 0.9 | 0.370 |
|  |  |  |  |  |  |  |  |  | |  |  |  |  |
|  |  |  | **t(9;11)** | | |  |  |  |  | |  | **Total** |  |
| KT analysed | 1791 |  | 1213 | 1192 | 5876 |  | 1893 | 4372 | |  |  | 14546 |  |
| t(9;11), % | 0.8 | NA | 2.2 | 1 | 1 |  | 0.7 | 0.8 | | NA | NA |  |  |
| Weight |  |  | 0.08 | 0.08 | 0.4 |  | 0.1 | 0.3 | |  |  |  |  |
| Proportion | 0.8 (0.4, 1.2) |  | 0.2 | 0.08 | 0.4 |  | 0.09 | 0.2 | |  |  | 1.0 | 0.395 |

| **Additional file 6: Supplementary Table 6: Raw data and statistical analysis for comparison with reports from the West contd..** | | | | | | | | | | | | | |
| --- | --- | --- | --- | --- | --- | --- | --- | --- | --- | --- | --- | --- | --- |
| **Country** | **This study**  **% (95% CI)** | **Germany** | **USA** | **UK** | | **Spain** | **Sweden** | **Germany** | **Australia** | | | **Total size &**  **weighted**  **proportions** |  |
|  |  | **Bacher** | **Byrd** | **Sanderson** | **Grimwade** | **Spain** | **Lazarevic** | **Creutzig** | **Nakase** | | **Gangatharan** |  |  |
|  |  |  | **All 11q23 abnormalities** | | |  |  |  |  | |  |  | **P value** |
| KT analysed | 1791 | 2235 | 1213 | 1192 | 5876 | 1129 | 1893 | 4372 | 230 | | 710 | 18850 |  |
| All 11q23q, % | 2.4 | 2.7 | 4.3 | 2 | 4 | 3.3 | 1.8 | 2.3 | 2.6 | | 3.5 |  |  |
| Weight |  | 0.1 | 0.06 | 0.06 | 0.3 | 0.06 | 0.1 | 0.2 | 0.01 | | 0.04 |  |  |
| Proportion | 2.4(1.7, 3.1) | 0.3 | 0.3 | 0.1 | 1.2 | 0.2 | 0.2 | 0.5 | 0.03 | | 0.1 | 3 | 0.137 |
|  |  |  |  | | |  |  |  |  | |  |  |  |
|  |  |  | **t(15;17)** | | |  |  |  |  | |  |  |  |
| KT analysed | 1791 |  | 1213 | 1192 | 5876 | 1129 |  | 4372 | 230 | | 710 | 14722 |  |
| t(15;17), % | 16.7 | NA | 6.7 | 8 | 13 | 14.8 | NA | 5.2 | 11.7 | | 7.8 |  |  |
| Weight |  |  | 0.08 | 0.08 | 0.4 | 0.08 |  | 0.3 | 0.02 | | 0.05 |  |  |
| Proportion | 16.7 (15.0, 18.4) |  | 0.5 | 0.6 | 5.2 | 1.1 |  | 1.5 | 0.2 | | 0.4 | 9.6 | <0.001 |
|  |  |  |  |  |  |  |  |  |  | |  |  |  |
|  |  |  | **inv 16** | | |  |  |  | |  |  |  |  |
| KT analysed | 1791 |  | 1213 | 1192 | 5876 | 1129 | 1893 | 4372 | 230 | | 710 | 16615 |  |
| inv 16, % | 1.7 | NA | 7.9 | 2 | 5 | 2.7 | 2.2 | 2.5 | 6.1 | | 1.9 |  |  |
| Weight |  |  | 0.07 | 0.07 | 0.4 | 0.07 | 0.1 | 0.3 | 0.01 | | 0.04 |  |  |
| Proportion | 1.7 (1.1, 2.3) |  | 0.6 | 0.1 | 1.8 | 0.2 | 0.3 | 0.7 | 0.08 | | 0.08 | 3.7 | <0.001 |
|  |  |  |  |  |  |  |  |  |  | |  |  |  |
|  |  |  | **Minus 17/abn 17p** | | |  |  |  | |  |  |  | |
| KT analysed | 1791 | 2235 | 1213 |  | 5876 |  | 1893 |  |  | |  | 11217 |  |
| Minus 17/abn 17p, % | 5.2 | 0.2 | 5.1 | NA | 4 | NA | 8.8 | NA | NA | | NA |  |  |
| Weight |  | 0.2 | 0.1 |  | 0.5 |  | 0.2 |  |  | |  |  |  |
| Proportion | 5.2 (4.2, 6.2) | 0.04 | 0.6 |  | 2.1 |  | 1.5 |  |  | |  | 4.2 | 0.035 |
|  |  |  | **Plus 21** | | |  |  |  |  | |  |  |  |
| KT analysed | 1791 | 2235 | 1213 |  | 5876 |  |  |  |  | | 710 | 10034 |  |
| Plus 21, % | 4.6 | 0.8 | 2.3 |  | 3 |  |  |  |  | | 0.7 |  |  |
| Weight |  | 0.2 | 0.1 |  | 0.6 |  |  |  |  | | 0.07 |  |  |
| Proportion | 4.6 (3.6, 5.6) | 0.2 | 0.3 |  | 1.8 |  |  |  |  | | 0.05 | 2.3 | <0.001 |

| **Additional file 6: Supplementary Table 6: Raw data and statistical analysis for comparison with reports from the West contd..** | | | | | | | | | | | | |
| --- | --- | --- | --- | --- | --- | --- | --- | --- | --- | --- | --- | --- |
| **Country** | **This study**  **% (95% CI)** | **Germany** | **USA** | **UK** | | **Spain** | **Sweden** | **Germany** | **Australia** | | **Total size &**  **weighted**  **proportions** |  |
|  |  | **Bacher** | **Byrd** | **Sanderson** | **Grimwade** | **Spain** | **Lazarevic** | **Creutzig** | **Nakase** | **Gangatharan** |  |  |
|  |  |  | **Complex KT (≥3 abnormalities)** | | |  |  |  |  |  |  | **P value** |
| KT analysed | 1791 | 2235 | 1213 | 1192 | 5876 | 1129 | 1893 | 4372 |  | 710 | 18620 |  |
| Complex (≥3), % | 15.6 | 12.3 | 11.1 | 15 | 14 | 19 | 24 | 14.5 |  | 20.6 |  |  |
| Weight |  | 0.1 | 0.07 | 0.06 | 0.3 | 0.06 | 0.1 | 0.2 |  | 0.04 |  |  |
| Proportion | 15.6 (13.9, 17.3) | 1.5 | 0.7 | 1.0 | 4.4 | 1.2 | 2.4 | 3.4 |  | 0.8 | 15.4 | 0.815 |
| KT, karyotype; Abn, abnormality. | | | | | | | | | | | | |
