# Supplementary material for: Cytogenetic profile of 1791 adult acute myeloid leukemia in India
Source: Mol Cytogenet. 2023 Sep 16;16:24. doi: 10.1186/s13039-023-00653-1 (PMC10504794; doi:10.1186/s13039-023-00653-1)
Supplement: Supplementary file 7 — Additional file 7. Raw data and statistical analysis for comparison of our findings with reports from S.E Asia. [file 13039_2023_653_MOESM7_ESM.docx]

| **Additional file 7: Supplementary Table 7: Raw data and statistical analysis for comparison of our findings with reports from S.E Asia.** | | | | | | | | | | | | | |
| --- | --- | --- | --- | --- | --- | --- | --- | --- | --- | --- | --- | --- | --- |
| **Country** | **This study % (95% CI)** | | **Japan** | | **Singapore** | **China** | | **Hong Kong** | **Korea** | **Malaysia** | | **Total size & weighted**  **proportions** |  |
|  |  |  | **Nakase** | **Wakui** | **Enjeti** | **Cheng** | **Li** | **So** | **Byun** | **Meng** | **Ambayya** |  |  |
|  |  | |  | **Normal karyotypes** | | |  |  |  |  |  |  | **P value** |
| KT analysed | 1791 | | 436 | 638 | 454 | 1293 | 2308 | 629 | 2132 | 480 | 601 | 8971 |  |
| Normal KT, % | 36.1 | | 43 | 42 | 39 | 42.3 | 39.8 | 39 | 42 | 70 | 49 |  |  |
| Weight |  | | 0.05 | 0.07 | 0.05 | 0.1 | 0.3 | 0.07 | 0.2 | 0.05 | 0.07 |  |  |
| Proportion | 36.1 (33.9, 38.3) | | 2.1 | 3.0 | 2.0 | 6.1 | 10.2 | 2.7 | 10.0 | 3.7 | 3.3 | 43.1 | <0.001 |
|  |  | |  |  |  |  |  |  |  |  |  |  |  |
|  |  | |  | **Abnormal karyotypes** | | |  |  |  |  |  |  |  |
| KT analysed | 1791 | | 436 | 638 | 454 | 1293 | 2308 | 629 | 2132 | 480 | 601 | 8971 |  |
| Abnormal KT, % | 64 | | 57 | 58 | 61 | 57.7 | 60.2 | 61 | 58 | 30 | 51 |  |  |
| Weight |  |  | 0.05 | 0.07 | 0.05 | 0.1 | 0.3 | 0.07 | 0.2 | 0.05 | 0.07 |  |  |
| Proportion | 64(61.8, 66.2) | | 2.8 | 4.1 | 3.1 | 8.3 | 15.5 | 4.3 | 13.8 | 1.6 | 3.4 | 56.9 | <0.001 |
|  |  | |  |  |  |  |  |  |  |  |  |  |  |
|  |  | |  | **inv(3) /t(3;3)** | | |  |  |  |  |  |  |  |
| KT analysed | 1791 | |  | 638 | 454 |  | 2308 | 629 | 2132 |  |  | 6161 |  |
| inv(3)/t(3;3), % | 1.8 | |  | 0.8 | 0.7 | NA | 0.6 | 1.2 | 0.4 | NA | NA |  |  |
| Weight |  | |  | 0.1 | 0.07 |  | 0.4 | 0.1 | 0.3 |  |  |  |  |
| Proportion | 1.8 (1.2, 2.4) | |  | 0.08 | 0.05 |  | 0.2 | 0.1 | 0.1 |  |  | 0.6 | <0.001 |
|  |  | |  |  |  |  |  |  |  |  |  |  |  |
|  |  | |  | **Del(5q)/minus 5** | | |  |  |  |  |  |  |  |
| KT analysed | 1791 | |  | 638 | 454 | 1293 | 2308 |  | 2132 | 480 |  | 7305 |  |
| Del 5q/minus 5, % | 6.7 | | NA | 0.3 | 6.6 | 0.8 | 1.7 | NA | 3.4 | 0.8 | NA |  |  |
| Weight |  | |  | 0.09 | 0.06 | 0.2 | 0.3 |  | 0.3 | 0.07 |  |  |  |
| Proportion | 6.7 (5.5, 7.9) | |  | 0.03 | 0.4 | 0.1 | 0.5 |  | 1.0 | 0.05 |  | 2.2 | <0.001 |
|  |  | |  |  |  |  |  |  |  |  |  |  |  |
|  |  | |  | **t(6;9)** | | |  |  |  |  |  |  |  |
| KT analysed | 1791 | |  | 638 | 454 |  |  | 629 | 2132 |  |  | 3853 |  |
| t(6;9), % | 0.9 | |  | 0.6 | 0.7 | NA | NA | 0.3 | 0.8 | NA | NA |  |  |
| Weight |  | |  | 0.2 | 0.1 |  |  | 0.2 | 0.6`` |  |  |  |  |
| Proportion | 0.9 (0.4, 1.3) | |  | 0.1 | 0.08 |  |  | 0.05 | 0.4 |  |  | 0.7 | 0.31 |
|  |  | |  |  |  |  |  |  |  |  |  |  |  |
| **Additional file 7: Supplementary Table 7: Raw data and statistical analysis for comparison of our findings with reports from S.E Asia contd..** | | | | | | | | | | | | | |
|  | **This study**  **% (95% CI)** | | **Japan** | | **Singapore** | **China** |  | **Hong Kong** | **Korea** | **Malaysia** | | **Total size & weighted**  **proportions** |  |
|  |  |  | **Nakase** | **Wakui** | **Enjeti** | **Cheng** | **Li** | **So** | **Byun** | **Meng** | **Ambayya** |  |  |
|  |  | |  | **Minus 7/del(7q)** | | |  |  |  |  |  |  | **P value** |
| KT analysed | 1791 | |  | 638 | 454 | 1293 | 2308 |  | 2132 | 480 |  | 7305 |  |
| Minus 7/del 7q, % | 9.3 | | NA | 0.3 | 7 | 1.4 | 2.7 | NA | 4.6 | 1.2 | NA |  |  |
| Weight |  | |  | 0.09 | 0.06 | 0.2 | 0.3 |  | 0.3 | 0.07 |  |  |  |
| Proportion | 9.3 (8.0, 10.6) | |  | 0.03 | 0.4 | 0.2 | 0.9 |  | 1.3 | 0.08 |  | 3.0 | <0.001 |
|  |  | |  |  |  |  |  |  |  |  |  |  |  |
|  |  | |  | **Plus 8** | | |  |  |  |  |  |  |  |
| KT analysed | 1791 | |  |  | 454 | 1293 | 2308 | 629 | 2132 | 480 | 601 | 7897 |  |
| Plus 8, % | 11.6 | | NA | NA | 7.3 | 3.8 | 5.5 | 4 | 7 | 3 | 3.2 |  |  |
| Weight |  | |  |  | 0.06 | 0.2 | 0.3 | 0.08 | 0.3 | 0.06 | 0.08 |  |  |
| Proportion | 11.6 (10.1, 13.1) | |  |  | 0.4 | 0.6 | 1.6 | 0.3 | 1.9 | 0.2 | 0.2 | 5.3 | <0.001 |
|  |  | |  |  |  |  |  |  |  |  |  |  |  |
|  |  | |  | **t(8;21)** | | |  |  |  |  |  |  | |
| KT analysed | 1791 | | 436 | 638 | 454 | 1293 | 2308 | 629 | 2132 | 480 | 601 | 8971 |  |
| t(8;21), % | 7.2 | | 13.2 | 17.7 | 7.5 | 8.3 | 15.1 | 9 | 9.7 | 7.5 | 8.5 |  |  |
| Weight |  | | 0.05 | 0.07 | 0.05 | 0.1 | 0.3 | 0.07 | 0.2 | 0.05 | 0.07 |  |  |
| Proportion | 7.2 (6.0, 8.4) | | 0.6 | 1.3 | 0.4 | 1.2 | 3.9 | 0.6 | 2.3 | 0.4 | 0.6 | 11.3 | <0.001 |
|  |  | |  |  |  |  |  |  |  |  |  |  |  |
|  |  |  |  | **t(9;22)** | | |  |  |  |  |  |  |  |
| KT analysed | 1791 | |  | 638 |  | 1293 | 2308 | 629 |  |  |  | 4868 |  |
| t(9;22), % | 1.1 | | NA | 1.1 | NA | 1.8 | 1.5 | 0.2 | NA | NA | NA |  |  |
| Weight |  | |  | 0.1 |  | 0.3 | 0.5 | 0.1 |  |  |  |  |  |
| Proportion | 1.1 (0.6, 1.6) | |  | 0.1 |  | 0.5 | 0.7 | 0.03 |  |  |  | 1.4 | 0.280 |
|  |  | |  |  |  |  |  |  |  |  |  |  |  |
|  |  | |  | **t(9;11)** | | |  |  |  |  |  |  | |
| KT analysed | 1791 | |  | 638 | 454 |  | 2308 | 629 | 2132 |  |  | 6161 |  |
| t(9;11), % | 0.8 | | NA | 1.6 | 0.9 | NA | 0.3 | 0.8 | 0.8 | NA | NA |  |  |
| Weight |  | |  | 0.1 | 0.07 |  | 0.4 | 0.1 | 0.3 |  |  |  |  |
| Proportion | 0.8 (0.4, 1.2) | |  | 0.2 | 0.07 |  | 0.1 | 0.08 | 0.3 |  |  | 0.7 | 0.612 |
|  |  | |  |  |  |  |  |  |  |  |  |  |  |
| **Additional file 7: Supplementary Table 7: Raw data and statistical analysis for comparison of our findings with reports from S.E Asia contd…** | | | | | | | | | | | | | |
|  | **This study**  **% (95% CI)** | | **Japan** | | **Singapore** | **China** | | **Hong Kong** | **Korea** | **Malaysia** | | **Total size & weighted**  **proportions** |  |
|  |  |  | **Nakase** | **Wakui** | **Enjeti** | **Cheng** | **Li** | **So** | **Byun** | **Meng** | **Ambayya** |  |  |
|  |  | |  | **All t(v;11q23)** | | |  |  |  |  |  |  | **P value** |
| KT analysed | 1791 | | 436 | 638 | 454 | 1293 | 2308 | 629 | 2132 |  | 601 | 8491 |  |
| All t(v;11q23), % | 2.4 | | 2.3 | 5 | 2.2 | 1.2 | 1.6 | 1.4 | 2.6 | NA | 2.5 |  |  |
| Weight |  | | 0.05 | 0.08 | 0.05 | 0.2 | 0.3 | 0.07 | 0.3 |  | 0.07 |  |  |
| Proportion | 2.4 (1.7, 3.1) | | 0.1 | 0.4 | 0.1 | 0.2 | 0.4 | 0.1 | 0.7 |  | 0.2 | 2.2 | 0.564 |
|  |  | |  |  |  |  |  |  |  |  |  |  | |
|  |  | |  | **t(15;17)** | | |  |  |  |  |  |  |  |
| KT analysed | 1791 | | 436 |  | 454 | 1293 | 2308 | 629 | 2132 | 480 | 601 | 8333 |  |
| t(15;17), % | 16.7 | | 11.2 | NA | 11 | 14.3 | 16.7 | 16 | 10.7 | 2.3 | 14.3 |  |  |
| Weight |  | | 0.05 |  | 0.05 | 0.2 | 0.3 | 0.08 | 0.3 | 0.06 | 0.07 |  |  |
| Proportion | 16.7 (15.0, 18.4) | | 0.6 |  | 0.6 | 2.2 | 4.6 | 1.2 | 2.7 | 0.1 | 1.0 | 13.1 | 0.0001 |
|  |  | |  |  | | |  |  |  |  |  |  |  |
|  |  | |  | **inv 16** | | |  |  |  |  |  |  |  |
| KT analysed | 1791 | | 436 | 638 | 454 |  | 2308 | 629 | 2132 |  | 601 | 7198 |  |
| inv(16)/t(16;16), % | 1.7 | | 2.7 | 4.1 | 1.1 | NA | 2.1 | 3 | 4.2 | NA | 5 |  |  |
| Weight |  | | 0.06 | 0.09 | 0.06 |  | 0.3 | 0.09 | 0.3 |  | 0.08 |  |  |
| Proportion | 1.7 (1.1, 2.3) | | 0.2 | 0.4 | 0.07 |  | 0.7 | 0.3 | 1.2 |  | 0.4 | 3.2 | **<0.001** |
|  | | | | | | | | | | | | | |
|  |  | |  | **Minus 17/abn(17p**) | | |  |  |  |  |  |  |  |
| KT analysed | 1791 | |  |  |  |  | 2308 |  |  |  |  | 2308 |  |
| Minus 17/abn 17p,  % | 5.2 (4.2, 6.2) | | NA | NA | NA | NA | 1.8 | NA | NA | NA | NA | 1.8 | <0.001 |
|  |  | |  |  |  |  |  |  |  |  |  |  |  |
| Weight |  | |  |  |  |  |  |  |  |  |  | 1.8 |  |
| Proportion | 5.2 (4.2, 6.2) | |  |  |  |  |  |  |  |  |  | 1.8 |  |
|  |  | |  |  |  | | |  |  |  |  |  |  |
|  |  | |  |  | **Plus 21** | | |  |  |  |  |  |  |
| KT analysed | 1791 | |  |  |  | 1293 | 2308 | 629 |  |  |  | 4230 |  |
| Plus 21, % | 4.6 | | NA | NA | NA | 1.6 | 2.4 | 1 | NA | NA | NA |  | |
| Weight |  | |  |  |  | 0.3 | 0.5 | 0.1 |  |  |  |  |  |
| Proportion | 4.6 (3.6, 5.6) | |  |  |  | 0.5 | 1.3 | 0.1 |  |  |  | 1.9 | <0.001 |
| **Additional file 7: Supplementary Table 7: Raw data and statistical analysis for comparison of our findings with reports from S.E Asia contd…** | | | | | | | | | | | | | |
|  | **This study**  **% (95% CI)** | | **Japan** | | **Singapore** | **China** | | **Hong Kong** | **Korea** | **Malaysia** | | **Total size & weighted**  **proportions** |  |
|  |  |  | **Nakase** | **Wakui** | **Enjeti** | **Cheng** | **Li** | **So** | **Byun** | **Meng** | **Ambayya** |  |  |
|  |  | |  |  | | |  |  |  |  |  |  | **P value** |
|  |  | |  | **Complex (≥3 abnormalities)** | | |  |  |  |  |  |  |  |
| KT analysed | 1791 | |  | 638 | 454 | 1293 | 2308 | 629 | 2132 | 480 |  | 7934 |  |
| Complex (≥3), % | 15.6 | | NA | 6.4 | 17 | 6.4 | 8.6 | 4 | 11.7 | 7.3 | NA |  |  |
| Weight |  | |  | 0.08 | 0.06 | 0.2 | 0.3 | 0.08 | 0.3 | 0.06 |  |  |  |
| Proportion | 15.6 (13.9, 17.3) | |  | 0.5 | 1.0 | 1.0 | 2.5 | 0.3 | 3.1 | 0.4 |  | 8.9 | <0.001 |
|  |  | |  |  |  |  |  |  |  |  |  |  |  |
|  |  | |  |  |  |  |  |  |  |  |  |  |  |
| CI, confidence interval; KT, karyotype; Abn, abnormality. | | | | | | | | | | | | | |
