# Supplementary material for: Cytogenetic profile of 1791 adult acute myeloid leukemia in India
Source: Mol Cytogenet. 2023 Sep 16;16:24. doi: 10.1186/s13039-023-00653-1 (PMC10504794; doi:10.1186/s13039-023-00653-1)
Supplement: Supplementary file 8 — Additional file 8. Raw data and statistical analysis for comparison with reports from N.Africa. [file 13039_2023_653_MOESM8_ESM.docx]

| **Additional file 8: Supplementary Table 8: Raw data and statistical analysis for comparison with reports from N.Africa.** | | | | | | | | | | | |
| --- | --- | --- | --- | --- | --- | --- | --- | --- | --- | --- | --- |
| **Country** | **This study**  **% (95% CI)** | **Tunisia** | | **Morocco** | | | **Egypt** | | **Total size & weighted** |  |  |
|  |  | **Gmidene** | | **Khoubila** | | | **ElNaggar** | |  |  |  |
|  |  | **Normal karyotypes** | | | | | | | **proportions** | **P value** |  |
| KT analysed | 1791 | 631 | | 895 | | | 120 | | 1646 |  |  |
| Normal KT, % | 36.1 | 37.1 | | 42 | | | 56.7 | |  |  |  |
| Weight |  | 0.4 | | 0.5 | | | 0.07 | |  |  |  |
| Proportion | 36.1 (33.9, 38.3) | 14.2 | | 22.8 | | | 4.1 | | 41.2 | <0.001 |  |
|  |  |  | |  | | |  | |  |  |  |
|  |  | **Abnormal karyotypes** | | | | | | |  |  |  |
| KT analysed | 1791 | 631 | | 895 | | | 120 | | 1646 |  |  |
| Abnormal KT, % | 64 | 62.9 | | 58 | | | 43.3 | |  |  |  |
| Weight |  | 0.4 | | 0.5 | | | 0.07 | |  |  |  |
| Proportion | 64(61.8, 66.2) | 24.1 | | 31.5 | | | 3.2 | | 58.8 | <0.001 |  |
|  |  |  | |  | | |  | |  |  |  |
|  |  | **inv(3) /t(3;3)** | | | | | | |  |  |  |
| KT analysed | 1791 |  | | 895 | | | 120 | | 1015 |  |  |
| inv(3) /t(3;3), % | 1.8 | NA | | 0.6 | | | 1.6 | |  |  |  |
| Weight |  |  | | 0.9 | | | 0.1 | |  |  |  |
| Proportion | 1.8 (1.2, 2.4) |  | | 0.5 | | | 0.2 | | 0.7 | <0.001 |  |
|  |  |  | |  | | |  | |  |  |  |
|  |  | **Del(5q)/minus 5** | | | | | | |  |  |  |
| KT analysed | 1791 | NA | | 895 | | |  | | 895 |  |  |
| Del 5q/minus 5, % | 6.7 |  | | 0.5 | | |  | | 0.5 | <0.001 |  |
| Proportion | 6.7 (5.5, 7.9) |  | |  | | |  | |  |  |  |
|  |  | **t(6;9)** | | | | | | |  |  |  |
| KT analysed |  | NA | | NA | | | NA | |  |  |  |
|  |  |  | |  | | |  | |  |  |  |
|  |  | **Minus 7/del(7q)** | | | | | | |  |  |  |
| KT analysed | 1791 | 631 | | 895 | | | 120 | | 1646 |  |  |
| Minus 7/del 7q, % | 9.3 | 3 | | 2.9 | | | 0.8 | |  |  |  |
| Weight |  | 0.4 | | 0.5 | | | 0.07 | |  |  |  |
|  |  | 1.2 | | 1.6 | | | 0.06 | | 2.8 | <0.001 |  |
| **Additional file 8: Supplementary Table 8: Raw data and statistical analysis for comparison with reports from N.Africa contd…** | | | | | | | | | | | |
| **Country** | **This study**  **% (95% CI)** | **Tunisia** | | **Morocco** | | | **Egypt** | | **Total size & weighted**  **proportions** |  |  |
|  |  | **Gmidene** | | **Khoubila** | | | **ElNaggar** | |  |  |  |
|  |  | **Plus 8** | | | | | | |  | **P value** |  |
| KT analysed | 1791 | 631 | | 895 | | | 120 | | 1646 |  |  |
| Plus 8, % | 11.6 | 7 | | 4.5 | | | 3.3 | |  |  |  |
| Weight |  | 0.4 | | 0.5 | | | 0.1 | |  |  |  |
| Proportion | 11.6 (10.1, 13.1) | 2.7 | | 2.4 | | | 0.2 | | 5.4 | <0.001 |  |
|  |  |  | |  | | |  | |  |  |  |
|  |  | **t(8;21)** | | | | | | |  |  |  |
| KT analysed | 1791 | 631 | | 895 | | | 120 | | 1646 |  |  |
| t(8;21), % | 7.2 | 12.2 | | 12.5 | | | 7.5 | |  |  |  |
| Weight |  | 0.4 | | 0.5 | | | 0.07 | |  |  |  |
| Proportion | 7.2 (6.0, 8.4) | 4.7 | | 6.8 | | | 0.5 | | 12.0 | <0.001 |  |
|  |  |  | |  | | |  | |  |  |  |
|  |  | **t(9;22)** | | | | | | |  |  |  |
| KT analysed | 1791 | NA | | NA | | | 120 | | 120 |  |  |
| t(9;22), % | 1.1 |  | |  | | | 0.8 | |  |  |  |
| Proportion | 1.1 (0.6, 1.6) |  | |  | | | 0.8 | | 0.8 | 0.154 |  |
|  |  |  | |  | | |  | |  |  |  |
|  |  | **t(9;11)** | | | | | | |  |  |  |
| KT analysed | 1791 |  | | 895 | | |  | | 895 |  |  |
| t(9;11), % | 0.8 |  | | 1 | | |  | |  |  |  |
| Proportion | 0.8 (0.4, 1.2) |  | | 1 | | |  | | 1 | 0.395 |  |
|  |  |  | |  | | |  | |  |  |  |
|  |  | **All t(v;11q23)** | | | | | | |  |  |  |
| KT analysed | 1791 | 631 | 895 | | | 120 | | | 1646 |  |  |
| All t(v;11q23), % | 2.4 | 3.8 | | 2.6 | | | 7.5 | |  |  |  |
| Weight |  | 0.4 | | 0.5 | | | 0.07 | |  |  |  |
| Proportion | 2.4 (1.7, 3.1 | 1.5 | | 1.4 | | | 0.5 | | 3.4 | 0.02 |  |
|  |  |  | | | | | | |  |  |  |
|  |  |  | | | | | | |  |  |  |
|  |  |  | | | | | | |  |  |  |
| **Additional file 8: Supplementary Table 8: Raw data and statistical analysis for comparison with reports from N.Africa contd…** | | | | | | | | | | | |
|  | **This study**  **% (95% CI)** | **Tunisia** | | | **Morocco** | | | **Egypt** | **Total size &**  **weighted**  **proportions** |  |  |
|  |  | **Gmidene** | | | **Khoubila** | | | **ElNaggar** |  |  |  |
|  |  | **t(15;17)** | | | | | | |  | **P value** |  |
| KT analysed | 1791 | 631 | | 895 | | | 120 | | 1646 |  |  |
| t(15;17), % | 16.7 | 13.2 | | 3.7 | | | 9.2 | |  |  |  |
| Weight |  | 0.4 | | 0.5 | | | 0.07 | |  |  |  |
| Proportion | 16.7 (15.0, 18.4) | 5.1 | | 2.0 | | | 0.7 | | 7.7 | <0.001 |  |
|  |  |  | |  | | |  | |  |  |  |
|  |  | **inv 16** | | | | | | |  |  |  |
| KT analysed | 1791 | 631 | | 895 | | | 120 | | 1646 |  |  |
| inv(16)/t(16;16), % | 1.7 | 3.5 | | 3.3 | | | 7.5 | |  |  |  |
| Weight |  | 0.4 | | 0.5 | | | 0.07 | |  |  |  |
| Proportion | 1.7 (1.1, 2.3) | 1.3 | | 1.8 | | | 0.5 | | 3.7 | <0.001 |  |
|  |  |  | |  | | |  | |  |  |  |
|  |  | **Minus 17/abn(17p)** | | | | | | |  |  |  |
| KT analysed | 1791 | NA | | NA | | | 120 | | 120 |  |  |
| Minus 17/17p abn, % | 5.2 |  | |  | | | 0.8 | | 0.8 | <0.001 |  |
| Proportion | 5.2 (4.2, 6.2) |  | |  | | |  | |  |  |  |
|  |  | **Plus 21** | | | | | | |  |  |  |
| KT analysed |  | NA | | NA | | | NA | |  |  |  |
|  |  |  | | | | | | |  |  |  |
|  |  | **Complex (≥3)** | | | | | | |  |  |  |
| KT analysed | 1791 | 631 | | 895 | | | 120 | | 1646 |  |  |
| Complex (≥3), % | 15.6 | 10.8 | | 7.4 | | | 0.8 | |  |  |  |
| Weight |  | 0.4 | | 0.5 | | | 0.07 | |  |  |  |
| Proportion | 15.6 (13.9, 17.3) | 4.1 | | 4.0 | | | 0.06 | | 8.2 | <0.001 |  |
| KT, karyotype; Abn, abnormality. | | | | | | | | | | |  |
